# Identification of small molecules targeting homoserine acetyl transferase from *Mycobacterium tuberculosis* and *Staphylococcus aureus*

**DOI:** 10.1038/s41598-022-16468-w

**Published:** 2022-08-13

**Authors:** Deepika Chaudhary, Avantika Singh, Mardiana Marzuki, Abhirupa Ghosh, Saqib Kidwai, Tannu Priya Gosain, Kiran Chawla, Sonu Kumar Gupta, Nisheeth Agarwal, Sudipto Saha, Yashwant Kumar, Krishan Gopal Thakur, Amit Singhal, Ramandeep Singh

**Affiliations:** 1 Translational Health Science and Technology Institute, NCR Biotech Science Cluster, Faridabad, Haryana 121001 India; 2 Manipal Academy of Higher Education, Manipal, Karnataka 576104 India; 3 Structural Biology Laboratory, Council of Scientific and Industrial Research-Institute of Microbial Technology, Chandigarh, 160036 India; 4 Infectious Diseases Labs (ID Labs), Agency for Science, Technology and Research (A*STAR), Singapore, 138648 Singapore; 5 Division of Bioinformatics, Bose Institute, Kolkata, West Bengal 700054 India; 6 Singapore Immunology Network (SIgN), (A*STAR), Singapore, 138648 Singapore; 7 Lee Kong Chian School of Medicine, Nanyang Technological University, Singapore, 308232 Singapore; 8 Tuberculosis Research Laboratory, NCR Biotech Science Cluster, 3rd Milestone, Faridabad-Gurugram Expressway, PO Box # 4, Faridabad, 121001 India

**Keywords:** High-throughput screening, Drug development

## Abstract

There is an urgent need to validate new drug targets and identify small molecules that possess activity against both drug-resistant and drug-sensitive bacteria. The enzymes belonging to amino acid biosynthesis have been shown to be essential for growth in vitro, in vivo and have not been exploited much for the development of anti-tubercular agents. Here, we have identified small molecule inhibitors targeting homoserine acetyl transferase (HSAT, MetX, Rv3341) from *M. tuberculosis*. MetX catalyses the first committed step in L-methionine and S-adenosyl methionine biosynthesis resulting in the formation of O-acetyl-homoserine. Using CRISPRi approach, we demonstrate that conditional repression of *metX* resulted in inhibition of *M. tuberculosis* growth in vitro. We have determined steady state kinetic parameters for the acetylation of L-homoserine by Rv3341. We show that the recombinant enzyme followed Michaelis–Menten kinetics and utilizes both acetyl-CoA and propionyl-CoA as acyl-donors. High-throughput screening of a 2443 compound library resulted in identification of small molecule inhibitors against MetX enzyme from *M. tuberculosis*. The identified lead compounds inhibited Rv3341 enzymatic activity in a dose dependent manner and were also active against HSAT homolog from *S. aureus*. Molecular docking of the identified primary hits predicted residues that are essential for their binding in HSAT homologs from *M. tuberculosis* and *S. aureus*. ThermoFluor assay demonstrated direct binding of the identified primary hits with HSAT proteins. Few of the identified small molecules were able to inhibit growth of *M. tuberculosis* and *S. aureus* in liquid cultures. Taken together, our findings validated HSAT as an attractive target for development of new broad-spectrum anti-bacterial agents that should be effective against drug-resistant bacteria.

## Introduction

Tuberculosis (TB) caused by *Mycobacterium tuberculosis* (*M. tuberculosis*) generally affects the lungs (pulmonary disease) but eventually disseminates in other organs resulting in extrapulmonary TB. The major challenges in the field of TB chemotherapy are (i) lengthy duration of chemotherapy, (ii) poor patient compliance and (iii) emergence of drug-resistant strains^1^. The recommended duration of treatment for individuals with drug-susceptible (DS) and drug-resistant (DR) TB lasts for 6-months and 18–24 months, respectively^2–4^. The cure rates of successful treatment of individuals with DS-TB and DR-TB are 85% and 55% respectively^5^. In order to tackle the global threat imposed by DR-TB, there is a need to validate novel drug targets, identify scaffolds that possess a novel mechanism, show compatibility with current first line TB drugs and are active against DR-*M. tuberculosis*^6^. The advent of genetic tools, sequencing platforms and screening methodologies have resulted in identification of various anti-tubercular agents with a novel mechanism of action^6^. Phenotypic and target based screening are mainstays of TB chemotherapy that have resulted in identification of various metabolic pathways as drug-targets^6,7^. Whole cell based assays have resulted in identification of several novel chemical entities that are currently being evaluated in different stages of clinical trials^8–14^. Majority of these new scaffolds inhibit enzymes involved in either cell wall biosynthesis or protein synthesis or energy production^6^. Among these, bedaquiline targeting ATP synthesis, delamanid and pretomanid targeting bacterial respiration and mycolic acid biosynthesis have been recently approved by FDA for treatment of individuals with DR-TB^15–17^.

Several studies have demonstrated that enzymes involved in amino acid biosynthesis are good drug-targets as humans lack a homolog for most of these targets^18–21^. Among 20 amino acids, phenylalanine, threonine, tryptophan, valine, isoleucine, methionine, leucine, histidine and lysine are considered to be essential for human growth and development. These essential amino acids can’t be synthesized de novo by humans and must be supplied in their daily diet^22^. In contrast, bacteria, plants and fungi encodes for enzymes involved in the biosynthesis of these amino acids. Therefore, targeting enzymes involved in amino acid biosynthesis might result in identification of highly specific anti-tubercular agents. Among the essential amino acids, L-threonine, L-lysine, L-methionine and L-isoleucine are derived from L-aspartate and constitute L-aspartate family amino acids (AFAA)^23^. The first committed step in AFAA biosynthesis is conversion of L-aspartate to aspartyl-4-phosphate by aspartokinase^24^. Subsequently, aspartate semialdehyde dehydrogenase converts aspartyl-4-phosphate to aspartyl-4-semialdehyde in a NADPH dependent manner^25^. Further, homoserine dehydrogenase (HSD) reduces aspartyl-4-semialdehyde to homoserine in a NADPH or NADH dependent manner^26,27^. The phosphorylation of homoserine by homoserine kinase is the first committed step in the biosynthesis of L-threonine or L-isoleucine^28^. The first committed step in L-methionine biosynthesis is the acetylation of homoserine by homoserine acetyl transferase (HSAT)^29^. This pathway also results in the meso-DAP synthesis, which is an important intermediate in cell wall and L-lysine biosynthesis^30^. Several studies have shown that *M. tuberculosis* strains with defects in biosynthesis of L-lysine or L-threonine or L-methionine are unable to grow in liquid cultures and establish infection in host tissues^31–33^. In agreement, *M. tuberculosis* strains with deletions in enzymes involved in biosynthesis of other essential amino acids are also attenuated for growth in vivo^31,34–36^.

Recently it has been demonstrated that killing of *M. tuberculosis* lacking HSAT, Rv3341, MetX is more rapid in comparison to other auxotrophic mutant strains in vitro^31^. Here, we have performed target based screening to identify small molecules that inhibit HSAT activity associated with MetX. These compounds were also able to bind and inhibit acetyl transferase activity associated with HSAT homolog from *S. aureus*. We identified HSAT inhibitors that were able to inhibit *M. tuberculosis* and *S. aureus* growth in vitro*.* Docking studies have identified amino acid residues that are involved in protein-small molecule interactions. Taken together, this is the first study where small molecules inhibitors have been identified against HSAT homologs from *M. tuberculosis* and *S. aureus*.

## Material and methods

### Chemicals, strains and growth conditions

The strains, plasmids and primers used in the study are shown in Table S1. The chemicals used in the present study were procured from Sigma, Merck unless mentioned. *Escherichia coli* XL-1 Blue and BL-21 (λDE3, plysS) were used for cloning and protein expression studies, respectively. *M. tuberculosis* H_37_Rv and *Staphylococcus aureus* (ATCC 25923) strains were used for growth inhibition experiments. In the present study, various *E.coli* and *S. aureus* (ATCC 25923) strains were cultured in Luria Bertani medium. The mycobacterial strains were cultured in Middlebrook 7H9 medium supplemented with 0.2% glycerol, 0.05% Tween-80, 1 × albumin dextrose saline (ADS) or Middlebrook 7H11 medium supplemented with 1 × oleic acid albumin dextrose saline (OADS) as previously described^37,38^. The antibiotics were used in the following concentration, kanamycin-25 µg/ml, tetracycline-10 µg/ml, ampicillin-50 µg/ml and chloramphenicol-34 µg/ml.

### Multiple sequence alignment of HSAT homologs

The amino acid sequences of prokaryotic HSAT homologs were retrieved from National Centre for Biotechnology Information Protein database. The multiple sequence alignment among protein sequences was performed using the Clustal Omega alignment tool and edited using GeneDoc^39^.

### Knocking down the expression of *metX* in *M. tuberculosis*

For knock down studies, the expression of MetX was silenced with the help of CRISPRi approach^40^. Briefly, the *metX*-specific guide sequence was generated by annealing complementary oligonucleotides and cloned adjacent to Cas9-handle sequence in the *E. coli*-mycobacteria shuttle plasmid pGrna resulting into pGrna*-metX*^40^. The recombinant pGrna-*metX* plasmid containing gene-specific guide sequence was transformed into *M. tuberculosis* harbouring a integrative copy of *dcas9* under the transcriptional control of anhydrotetracycline inducible promoter. The transformants were selected on MB7H11 plates supplemented with kanamycin and hygromycin. Simultaneously, *M. tuberculosis* harbouring dCas9 was transformed with empty plasmid pGrna and used as ‘Control’. The suppression of MetX expression in *metX* knock down strains was achieved by inducing the bacterial cultures with the addition of 50 ng/ml anhydrotetracycline (Atc).

### qPCR studies

Total RNA was extracted at different timepoints using Trizol method as per standardised protocols. The extracted RNA was treated with DNase I using Ambion DNA-free kit and subjected to cDNA synthesis using random hexamer primers and superscript reverse transcriptase III as per standardised protocols. qPCR was performed using the synthesized cDNA and gene specific primers in ABI 7500 fast real time-PCR machine as per manufacturer’s recommendations. The relative levels of *metX* were quantified in knock down strains after normalisation with housekeeping gene, *sigA* as previously described^38^.

### Expression and purification of HSAT proteins

For expression studies, gene encoding HSAT from either *M. tuberculosis* and *S. aureus* genome was PCR amplified and cloned into prokaryotic expression vector, pET28b. MetX^S157A^ point mutant from *M. tuberculosis* was generated by two-step PCR using gene specific primers harbouring the desired mutation. The sequence of all recombinant constructs was confirmed by sequencing. The recombinant constructs were transformed into BL-21 (λDE3, plysS) and protein expression was induced by the addition of 0.3 mM isopropyl β-D-1-thiogalactopyranoside (IPTG) at O.D_600nm_ ~ 0.5. The induced cultures were incubated overnight at 18 °C with constant shaking at 200 rpm, harvested by centrifugation and resuspended in lysis buffer (20 mM Tris–Cl, pH 8.0, 150 mM NaCl, 5 mM β-mercaptoethanol, 10% glycerol and 1 mM PMSF). The cells were lysed by sonication and cell debris was removed by centrifugation of the sonicated lysates at 12,000 g for 45 min. The supernatant was incubated with nickel-nitrilotriacetic acid (Ni–NTA) resin and protein purification was performed as per manufacturer’s recommendation. The recombinant protein was eluted using elution buffer (20 mM Tris pH 8.0, 200 mM NaCl, 5 mM β-mercaptoethanol) supplemented with the increasing concentrations of imidazole (10–500 µM). The selected purified fractions were concentrated and subjected to gel exclusion chromatography using Superdex 200 Increase 10/300 GL column (GE Healthcare). The purified fractions were pooled, dialysed, concentrated and stored as aliquots in enzyme storage buffer (50 mM NaH_2_PO_4_, 300 mM NaCl). The amount of protein in concentrated fractions was quantified using Bradford reagent as per standard protocols. The concentrated proteins were stored in − 80 °C till further use.

### Analytical ultracentrifugation experiments

In order to determine the oligomeric state of MetX, analytical ultracentrifugation experiments were performed using Beckman-Coulter XL-A analytical ultracentrifuge equipped with a TiAn50 eight hole rotor. Two-channel epon centrepieces and quartz windows were used in the experiment. The protein samples at three different concentrations (10, 20 and 30 μM in 20 mM Tris–Cl, pH 7.5 and 150 mM NaCl buffer) were prepared, centrifuged at 40,000 rpm and the absorbance scans were recorded at 280 nm every 3 or 4 min. To fit multiple scans at regular intervals, SEDFIT continuous distribution c(s) model was used^41^. SEDNTERP (http://www.rasmb.bbri.org/) was used to find the solvent viscosity (η) and density (ρ).

### Far ultra violet circular dichroism (CD) spectroscopy

CD spectra of wild type and mutant protein was recorded on a Jasco-J-815 spectropolarimeter in 10 mM sodium phosphate buffer pH-7.5. The protein concentration for CD-spectra analysis was kept at 3 µM and data was recorded at 25 °C. The spectra were recorded from 190 to 250 nm using 2-mm path length cuvette with a scan rate of 50 nm/min and averaged over ten scans. The raw CD data was converted into mean residue ellipticity at wavelength λ ([Φ]_MRWλ_) by using the following formula:

$$\mathrm{Mean}\,\mathrm{residue}\,\mathrm{ellipticity}\,[\Phi]_{MRW\uplambda}\,=\,\mathrm{MRWx}\Phi_{\uplambda}/10\mathrm{xcxd}$$

where, Φ_λ_ is observed ellipticity (degrees) at wavelength λ, MRW is mean residue weight, c is concentration in g/ml and d is path length in cm^42^.

### Biochemical characterization of MetX

To optimise the assay conditions for high through put screening, various parameters such as pH buffer, ion concentration, substrate specificity, and incubation time were standardised. The acetyl transferase activity was measured spectrophotometrically at 412 nm with the addition of 0.2 mM Ellman’s reagent (5,5’-thiobis-(2-nitrobenzoic acid, DTNB)^43^. For *K*_*m*_,* V*_*max*_,* k*_*cat*_*/K*_*m*_ determination, initial velocities in enzyme reactions (rate of CoA released in µM) were plotted against different concentration of acetyl-CoA using non-linear regression to the Michaelis–Menten equation. The substrate specificity of HSAT was also determined by performing enzymatic reactions in the presence of either 1.0 mM L-homoserine or L-serine or L-threonine. The enzymatic reactions were also performed using 1.0 mM L-homoserine in the presence of either 1.0 mM acetyl-CoA or propionyl-CoA or succinyl-CoA. For identification of residues essential for acetyl-transferase activity, enzyme assays were performed in standardised conditions using either 0.5 µM wild type or mutant MetX proteins.

### High throughput screening to identify MetX inhibitors

An endpoint HSAT activity assay was performed using the 2443 structurally diverse compounds belonging to small molecule library of the National Cancer Institute Developmental Therapeutic Program (NCI-DTP) in 96 well format. High throughput screening assays were performed in 30 µl reaction volume containing 100 mM Tris–Cl, pH-7.4, 5.0 mM MgCl_2_, 0.5 µM (His)_6_-Rv3341 and the corresponding compound of the chemical library at a single concentration of 100 µM. The enzyme scaffold mix was incubated at room temperature for 10 min and the reaction was initiated by the addition of 1.0 mM L-homoserine and 1.0 mM acetyl-CoA. The reaction was further incubated at 37 °C for 10 min, the amount of CoA released in enzymatic reaction was determined upon DTNB addition. All reaction plates contained appropriate no enzyme, no substrate and assay buffer only control. In all our enzymatic assays, the data obtained was corrected for spontaneous hydrolysis of CoA ester using the following formula:

$$\begin{aligned}&\mathrm{CoA}\,\mathrm{release}\,\mathrm{in}\,\mathrm{total}\,\mathrm{reaction}-(\mathrm{CoA}\,\mathrm{release}\,\mathrm{in}\,\mathrm{no}\,\mathrm{substrate}\,\mathrm{control}+\mathrm{CoA}\,\mathrm{release}\,\mathrm{in}\,\mathrm{no}\,\mathrm{enzyme}\,\mathrm{control}\\&\quad-\mathrm{CoA}\,\mathrm{release}\,\mathrm{in}\,\mathrm{buffer}\,\mathrm{only}\,\mathrm{control}).\end{aligned}$$

Subsequently, total activity and percentage inhibition was calculated for each compound.

### Determination of specificity and potency of the identified primary hits

Next, we evaluated the ability of the identified primary hits to inhibit HSAT activity associated with homologs from *M. tuberculosis* and *Staphylococcus aureus* (*S. aureus*). The inhibition assays for *S. aureus* homolog were performed in triplicates in conditions standardised for *M. tuberculosis* HSAT protein. The activity assays were also performed at different concentration of the primary hits in the range of 6.25 to 100 µM.

### Characterization of the binding of small molecule with HSAT protein using ThermoFluor assays

The SYPRO Orange dye was used to monitor protein folding in the absence or presence of primary hits. The thermal shift assays were performed in reaction buffer consisting of 5 µM of purified protein and SYPRO orange dye. The protein samples were heated from 20 to 95 °C with an increment of 1 °C per 1 min cycle. The increase in temperature results in protein unfolding, SYPRO Orange dye binds to unfolded protein and fluorescence intensity is measured at an excitation and emission wavelength of 372/472 nm and 570 nm, respectively. The data was acquired from step-one software and plotted to calculate melting temperature (T_m_) of HSAT in the absence or presence of different compounds.

### Molecular docking studies

For docking studies, the three-dimensional structures of HSAT homologs from *M. tuberculosis* (PDB id: 6PUX) and *S. aureus* (PDB id: 4QLO) were downloaded from PDB^44,45^. AutoDock-Tools were used for preparing the protein structures, determining the target regions for ligand binding and analysing the docking results^46,47^. Open Babel Platform was used to convert the file types of the ligands from SDF to PDBQT format^48^. A grid of 28 × 32 × 22 Å^3^ comprising the highly conserved catalytic triad (serine at 157, aspartic acid 320 and histidine 350) and the nearby surface groove was selected for docking of small molecules in *M. tuberculosis* MetX. Similarly, a grid of 20 × 20 × 20 Å^3^ accommodating the catalytic triad (serine at 131, aspartic acid 267 and histidine 296) and the surface groove in *S. aureus* HSAT was selected for docking^45^. The processing of input and output files of AutoDock Vina was achieved using in-house Perl scripts.

### Determination of the antibacterial activity of primary hits in liquid cultures and macrophages

The antimycobacterial activity of hits identified from high throughput screening was measured as previously described^37^. Briefly, the small molecules were serially diluted 2.0-fold in the range of 200–0.195 µM with a final volume of 50 µl. Subsequently early-logarithmic culture of *M. tuberculosis* H_37_Rv (OD_600nm_ ~ 0.2) was serially diluted 1:1000, 50 µl of diluted cultures was added to each well and plates were incubated at 37 °C for 14 days. The minimum inhibitory concentration (MIC_99_) was the lowest concentration of compound that completely inhibited *M. tuberculosis* growth in 96 well plates. For determining in vitro killing in *M. tuberculosis*, early-logarithmic cultures of (OD_600_ ~ 0.2) were exposed to 10 × MIC_99_ concentration of drugs for either 2, 4 or 7 days. For L-methionine supplementation experiments, *M. tuberculosis* was exposed to 5x-MIC_99_ concentration of NSC369066 in the absence or presence of 250 µg/ml L-methionine for 7 or 10 day. At designated time point, 10.0-fold serial dilutions were prepared, plated on MB7H11 supplemented with oleic acid–ADC (OADC) and incubated at 37 °C for 3–4 weeks. For determining in vitro killing of *S. aureus*, early-logarithmic cultures (OD_600nm_ ~ 0.3–0.4) were exposed to 100 µM of respective drugs and OD_600nm_ was determined at regular intervals. The determination of TC_50_ for primary hits and intracellular killing experiments in THP-1 macrophages were performed as previously described^49^.

### Statistics

GraphPad Prism version 8 (GraphPad Software Inc., CA, USA) was used for statistical analysis and the generation of graphs. All values are expressed as the mean ± SEM.

## Results

### Sequence homology of various prokaryotic HSAT homologs

**Figure 1:**
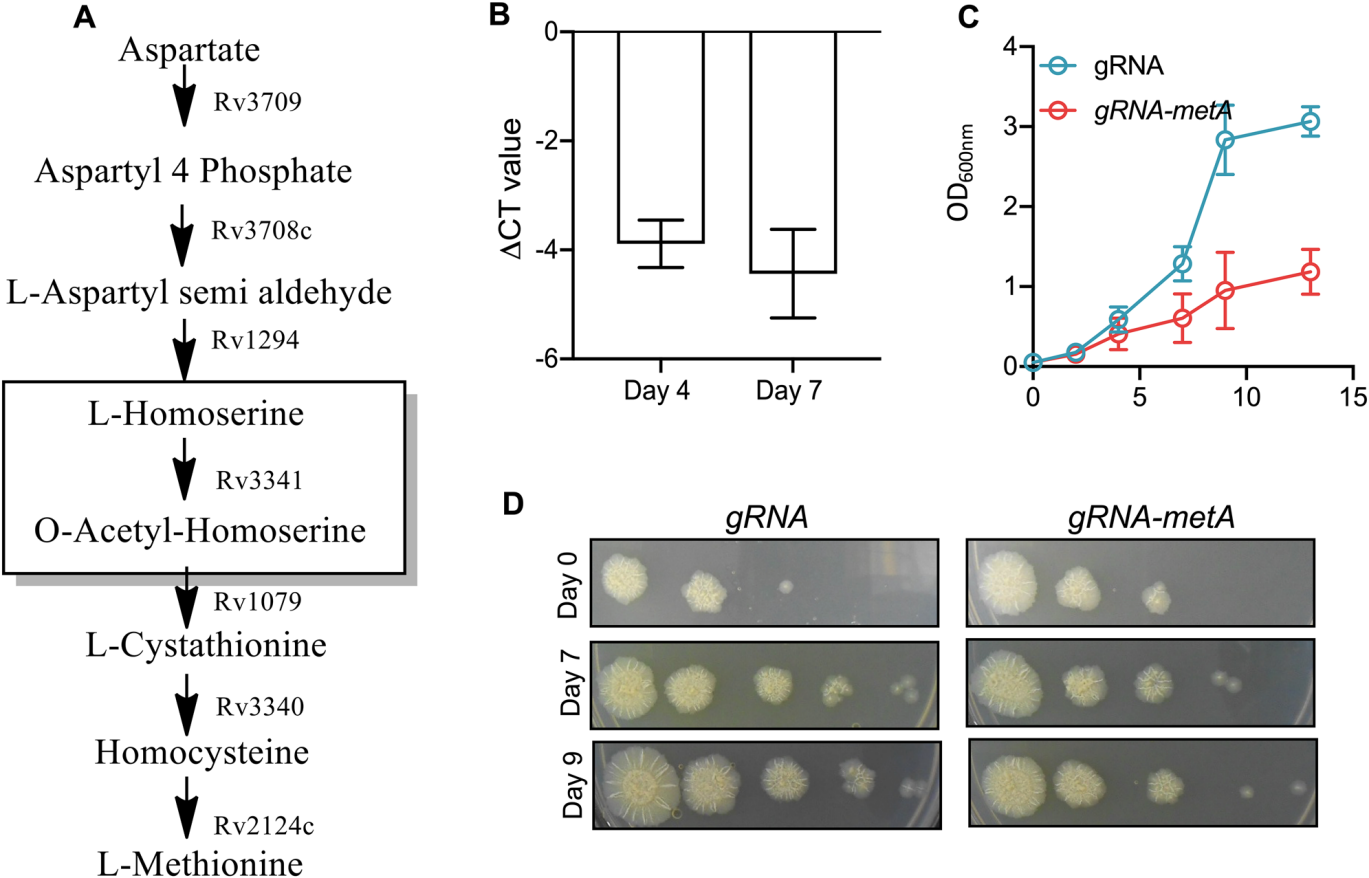
(**A**) Schematic representation of L-methionine biosynthetic pathway in *M. tuberculosis*. The enzymes involved in L-methionine biosynthetic pathway of *M. tuberculosis* are shown. Rv3341, highlighted in the box encodes for homoserine acetyl transferase that catalyses the first committed step in L-methionine biosynthesis. (**B**) Silencing of Rv3341 expression in *M. tuberculosis* using CRISPRi approach. The effect of CRISPRi on *metX* expression in knock down strain was quantified by qPCR using gene specific primers. The data shown in this panel is mean ± S.E. fold change of ΔCT obtained from two experiments performed in duplicates (**C**,**D**) Effect of *metX* repression on *M. tuberculosis* growth in vitro. The effect of conditional repression of *metX* on in vitro growth of *M. tuberculosis* was determined by measuring absorbance (OD_600nm_, **C**) at regular intervals. In addition, 10.0 fold serial dilutions of cultures were spotted on MB7H11 plates (**D**). The plates were incubated at 37 °C for 3–4 weeks. The data shown in panel C is mean ± S.E. of OD_600nm_ values obtained from three independent experiments. The data shown in panel D is representative of two independent experiments.

L-Methionine is an essential amino acid and is not synthesized de novo in humans. In plants, fungi and bacteria, L-methionine, L-threonine and L-lysine are derived from L-aspartate^23^. HSAT performs the first committed step in L-methionine biosynthesis and converts L-homoserine to o-acetyl homoserine using acetyl-CoA as an acetate donor (Fig. 1A). L-methionine is converted to S-adenosyl methionine (SAM) by methionine adenosyl transferase and SAM serves as a methyl donor in several methyl transferase reactions and is converted to S-adenosyl homocysteine^50,51^. L-methionine can also be regenerated from homocysteine by the enzyme methionine synthase in a Vitamin B12 dependent manner^52^. Multiple sequence alignment among HSAT homologs from various prokaryotes revealed that these proteins share significant sequence identity among themselves (Fig. S1). Among the known homologs from non-mycobacterial species, *M. tuberculosis* MetX protein shared the maximum identity of 48% with HSAT from *C. glutamicum* and 37% identity with homologs from *L. interrogans* and *H. influenzae* (Fig. S1). The most conserved sequence of HSAT protein is the catalytic triad that comprises of a hydrogen bonded residue such as a nucleophile, an acid and a conserved histidine residue^53^. This catalytic triad is also present in MetX protein from *M. tuberculosis* and the residues forming this catalytic triad are Ser157, Asp320 and His350 (Fig. S1).

### *M. tuberculosis* growth is reduced upon suppression of Rv3341 expression

We next sought to reverify the essentiality of MetX in *M. tuberculosis* growth using CRISPRi approach^40^. We cloned 20 bp sgRNA binding to complementary region of Rv3341 in Atc based expression vector, pGrna. The recombinant pGrna-*metX* was electroporated in *M. tuberculosis* strain harboring a single copy of *dcas9*. In our experiments the *dcas9* strain harboring empty pGrna construct was used as control. The expression of gRNA and *dcas9* was induced by the addition of 50 ng/ml Atc. qPCR studies using gene specific primers revealed that sgRNA-*dcas9* co-expression reduced the transcript levels of *metX* by ~ 15.0–20.0 folds at day 4 and day 7 post-Atc induction (Fig. 1B). We next compared the growth of *M. tuberculosis dcas9* strain harboring either pGrna or gRNA*-metX* by measuring OD_600nm_ at regular intervals and by spotting anhydrotetracycline induced *M. tuberculosis* cultures on MB7H11 plates. As expected, we noticed that *M. tuberculosis* growth was reduced upon repression of MetX in both liquid and solid medium at different time points post-induction (Fig. 1C). Taken together, these observations reconfirmed the essentiality of L-methionine biosynthetic pathway for *M. tuberculosis* growth in vitro^31^ (Fig. 1D).

### Rv3341 exists in three distinct oligomeric state

**Figure 2:**
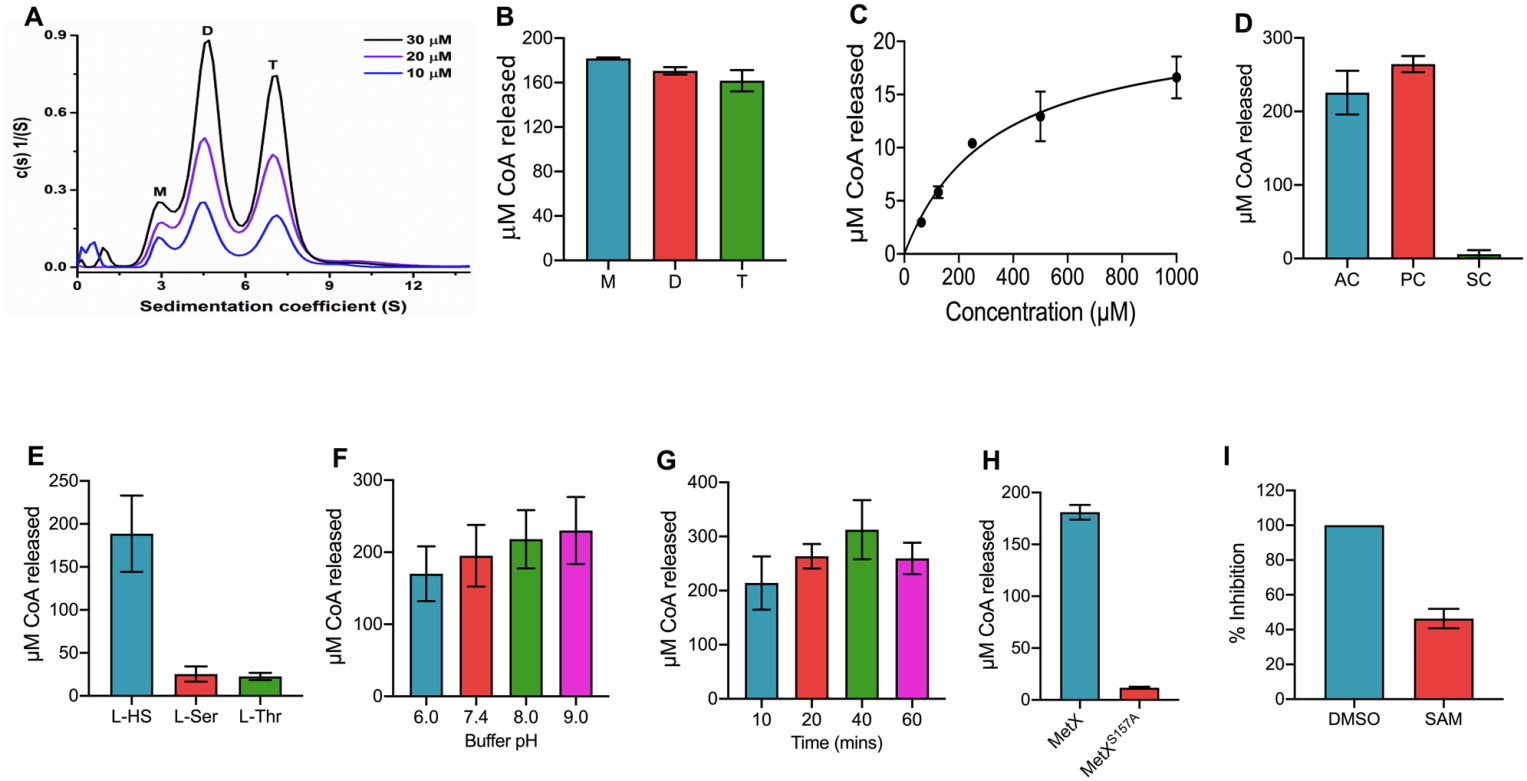
Biochemical characterization of Rv3341. (**A**) Oligomeric state of Rv3341 enzyme. Analytical ultracentrifugation was performed using purified (His)_6_-MetX at 10 µM, 20 µM and 30 µM concentration in 20 mM Tris–Cl buffer, pH-7.5 containing 150 mM NaCl. (**B**) Enzymatic activity of different oligomeric forms of Rv3341. The amount of CoA released in enzymatic reactions in the presence of 0.5 µM of monomer, dimer or tetramer form of Rv3341 was determined. (**C**) Michaelis Menten plot for Rv3341 enzymatic activity. The enzymatic reactions were performed in vitro using 1 mM L-homoserine and different concentrations of acetyl-CoA. (**D**,**E**) Substrate specificity of Rv3341 enzyme. In order to determine the substrate specificity of Rv3341 for acetyl-CoA (D), enzymatic reactions were performed in the presence of either 1.0 mM acetyl-CoA (AC) or propionyl-CoA (PC) or succinyl-CoA (SC). The specificity for L-homoserine was determined by performing enzymatic reaction in the presence of either 1.0 mM L-homoserine (L-HS) or L-serine (L-Ser) or L-threonine (L-Thr). (**F**) Effect of buffer pH on Rv3341 activity in vitro. HSAT activity assays were performed in buffers of pH in the range of 6.0–9.0. (**G**) Time kinetics analysis of Rv3341 activity. The HSAT activity was calculated after 10 min, 20 min, 40 min and 60 min post-incubation with substrates. (**H**) HSAT activity of wild type and mutant proteins. The enzymatic assays were performed using 0.5 µM of purified wild type and mutant protein in assay conditions containing 1.0 mM L-homoserine and 1.0 mM acetyl-CoA. (**I**) Inhibition of Rv3341 activity by SAM. Rv3341 enzymatic activity was calculated in presence of 100 µM of SAM. The amount of coenzyme A released in the enzymatic reactions was determined upon the addition of Ellman reagent and measuring absorbance at 412 nm. The data shown on y-axis in panels B-H is µM CoA released in enzymatic reaction and values shown are mean ± S.E. obtained from three independent experiments performed in duplicates. The data shown in panel I is mean ± S.E. of percentage inhibition obtained from two independent experiments performed in duplicates.

The recombinant (His)_6_- MetX was purified to near homogeneity using Ni–NTA metal affinity chromatography followed by gel filtration. The purity of the protein in different fractions was determined using 15% SDS-PAGE and a single band with a molecular weight of ~ 43 kDa was observed after Coomassie brilliant blue staining. HSAT proteins from other organisms such as *Leptospira interrogans, Haemophilus influenzae, Staphylococcus aureus* have been reported to form dimers, thus, we next determined the oligomeric state of *M. tuberculosis* MetX protein in solution^45,53,54^. Three concentrations i.e. 10 µM, 20 µM and 30 µM of the purified Rv3341 protein were used for performing Sedimentation Velocity Analytical Ultracentrifugation (SV-AUC) experiments as described in Materials and Methods. Sedimentation velocity analysis of (His)_6_-MetX resulted in three peaks with apparent molecular weight of ~ 43 kDa, 82.5 kDa and 158 kDa (Fig. 2A). Thus, these observations suggest that MetX exists in multiple oligomeric states in solution such as monomer, dimer or tetramer.

### Determination of steady state kinetics parameters and optimisation of assay conditions

Next, various kinetic parameters of (His)_6_-MetX was determined by monitoring CoA production as described in Materials and Methods. We initially evaluated enzymatic activity associated with monomer, dimer and tetramer fractions of the purified protein. We observed that all three oligomeric states showed comparable in vitro activity resulting in the formation of approximately 160–180 µM CoA (Fig. 2B). We next performed steady state kinetics using 1.0 mM L-homoserine and different concentration of acetyl-CoA in the presence of 0.5 µM MetX enzyme. As shown in Fig. 2C, the formation of O-acetyl homoserine followed Michaelis–Menten kinetics with a *K*_*m*_ of 328 µM and *k*_*cat*_ of 44.12 min^−1^. The catalytic efficient constant (*k*_*cat*_*/K*_*m*_) for MetX was 0.134 µM^−1^ min^−1^. In order to determine substrate specificity, we next evaluated MetX activity in the presence of either acetyl-CoA, succinyl-CoA or propionyl-CoA. As shown in Fig. 2D in addition to acetyl-CoA, (His)_6_- MetX was also able to use propionyl-CoA as an acyl-donor. We observed that the activity obtained in the presence of propionyl-CoA was comparable to the activity observed in the presence of acetyl-CoA (Fig. 2D). However, the transferase activity seen in reactions containing succinyl-CoA as an acyl-donor was ~ 2.5% of that observed in enzyme assays performed in the presence of acetyl-CoA (Fig. 2D). We also determined substrate specificity of MetX enzyme for either L-homoserine or L-serine or L-threonine and found that (His)_6_- MetX prefers L-homoserine as substrate as approximately 10–13% activity was observed in the enzymatic reactions containing either L-serine or L-threonine as substrates (Fig. 2E).

The pH dependence of HSAT was also monitored over the range of pH 6.0–9.0 using 1.0 mM L-homoserine and 1.0 mM acetyl-CoA. The results obtained indicated that Rv3341 enzymatic activity varied little with changes in pH of the assay buffer (Fig. 2F). We also performed time kinetics and observed that maximal activity was observed within 10 min upon the addition of substrates (Fig. 2G). We did not observe any increase upon inclusion of 0.1% Triton-X-100 in the assay buffer of enzymatic reaction (data not shown). As expected, in comparison to the wild type protein, (His)_6_-MetX^S157A^ (Ser 157 belongs to catalytic triad) displayed activity of 7% relative to the wild type protein (Fig. 2H). Next, we performed CD spectroscopy experiments to determine whether the observed loss in activity of the mutant protein is associated with major secondary structural changes. The analysis of the data revealed that the CD spectra of wild type and MetX^S157A^ was comparable. These observations suggest that Ser157 is essential for its enzymatic activity, however, its mutation to alanine did not result in any major secondary structure changes (data not shown). Further, activity assays were also performed in the presence of SAM, a known inhibitor of MetX^55^. We observed approximately 60% reduction in the activity of HSAT activity in the presence of SAM (Fig. 2I). Taken together, we demonstrate that Rv3341 prefers L-homoserine and acetyl-CoA as substrates and Ser^157^ residue is essential for its enzymatic activity.

### High throughput screening against MetX enzyme using NCI-DTP library

**Figure 3:**
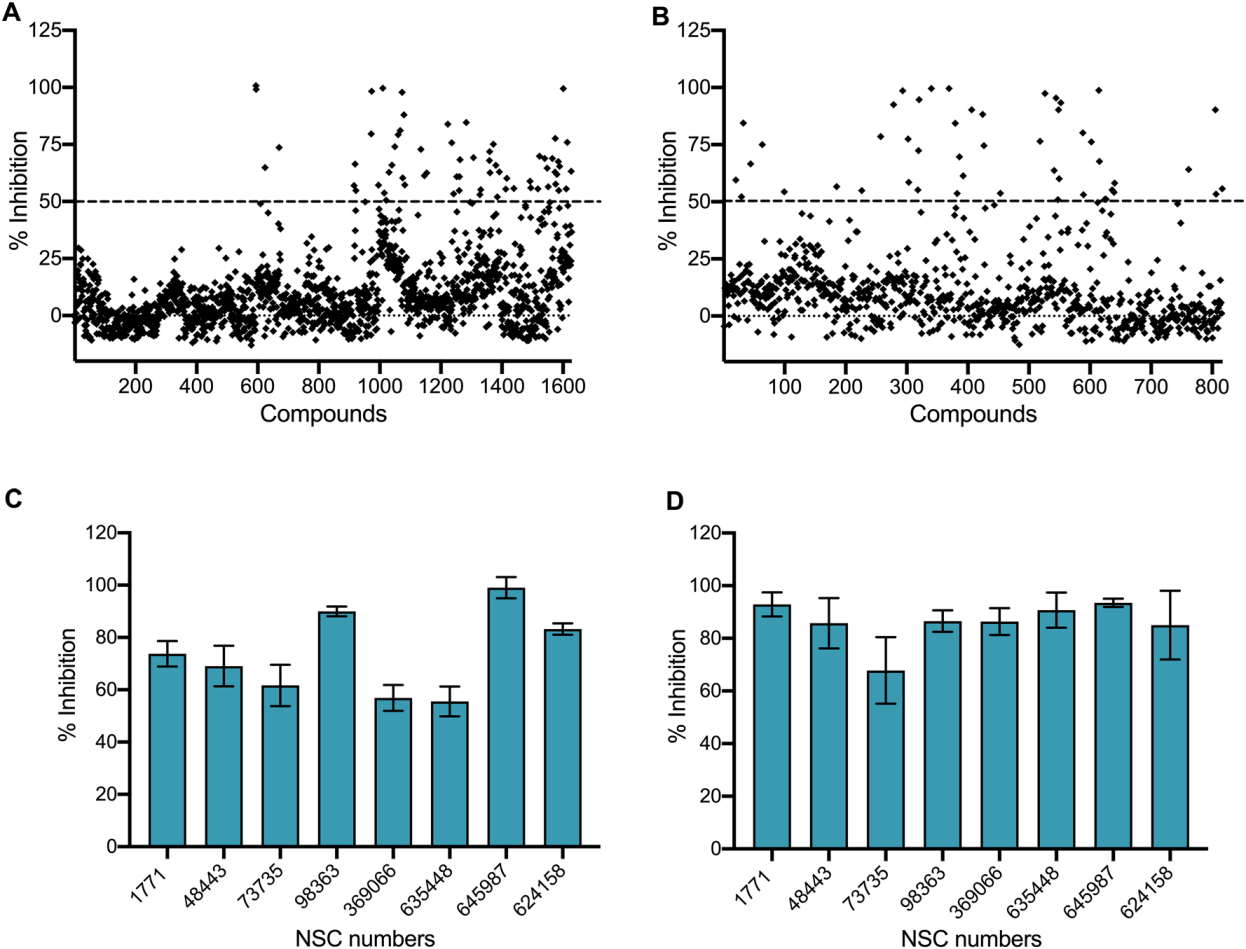
(**A**,**B**) Preliminary screening of HSAT activity using small molecules belonging to National Cancer Institute-Developmental Therapeutic Program Library. (**A**) The entire NCI-DTP library comprising of 2443 compounds belonging to either diversity set (**A**) and mechanistic set (B) was screened at 100 µM concentration to identify inhibitors for Rv3341. The data shown in this panel is percentage inhibition obtained from a single experiment. (**C**,**D**) HSAT enzymatic inhibition by the identified primary hits. The homoserine acetyl transferase activity associated with HSAT homologs from either *M. tuberculosis* (**C**) or *S. aureus* (**D**) was measured in the presence of identified primary hits at 100 µM concentration. The data shown in these panels is mean ± S.E. of percentage inhibition obtained from three independent experiments performed in duplicates.

Using the above standardised parameters, we performed a high-throughput screen (HTS) using a 2443 compound library to identify inhibitors for MetX activity in vitro. In our preliminary screen, 113 compounds were able to inhibit MetX enzymatic activity by more than 50% in vitro at 100 µM concentration (Fig. 3A,B, Table S2). Among these 65 and 48 small molecules belonged to diversity and mechanistic set, respectively. To eliminate false positives, inhibition assays were repeated using 26 primary hits. Among these, 8 compounds, NSC1771 (also known as Thiram), NSC48443, NSC73735, NSC98363, NSC369066, NSC635448, NSC645987 and NSC624158, inhibited MetX activity by more than 50% at 100 µM concentration (Fig. 3C).

We next assessed the activity of the above 8 compounds against HSAT of *Staphylococcus aureus,* a gram positive bacteria responsible to cause skin infections^56^. Due to the issue of drug-resistance (wide spread of methicillin resistant *S. aureus* strain) there is an urgency to develop new drugs against this human pathogen^57^. The genome of *S. aureus* encodes for a HSAT homolog that shares an identity of 28.4% with *M. tuberculosis* MetX (Fig. S1). It has been previously shown that HSAT protein from *Staphylococcus aureus* forms dimers and *k*_*cat*_/*K*_*m*_ values for acetyl-CoA and L-homoserine were observed to be 4 × 10^5^ and 3.3 × 10^5^ M^−1^ s^−1^, respectively^45^. The superimposition of MetX three-dimensional structure (PDB id: 6PUX) over the three-dimensional structure of HSAT protein from *S. aureus* (PDB code: 4QLO) resulted in r.m.s.d of 1.666 Å (Fig. S2A). Hence, we hypothesized that the identified small molecules might also be able to inhibit the activity associated with *S. aureus* homolog. The recombinant protein was purified, concentrated, dialysed and enzymatic assays were performed in conditions standardised for *M. tuberculosis* homolog. As shown in Fig. 3D, we observed that the identified primary hits inhibited enzymatic activity of the *S. aureus* homolog. The identified primary hits NSC1771, NSC48443, NSC73735, NSC98363, NSC369066, NSC635448, NSC645987 and NSC624158 inhibited the formation of CoA in enzymatic reactions by > 60% (Fig. 3D).

**Figure 4:**
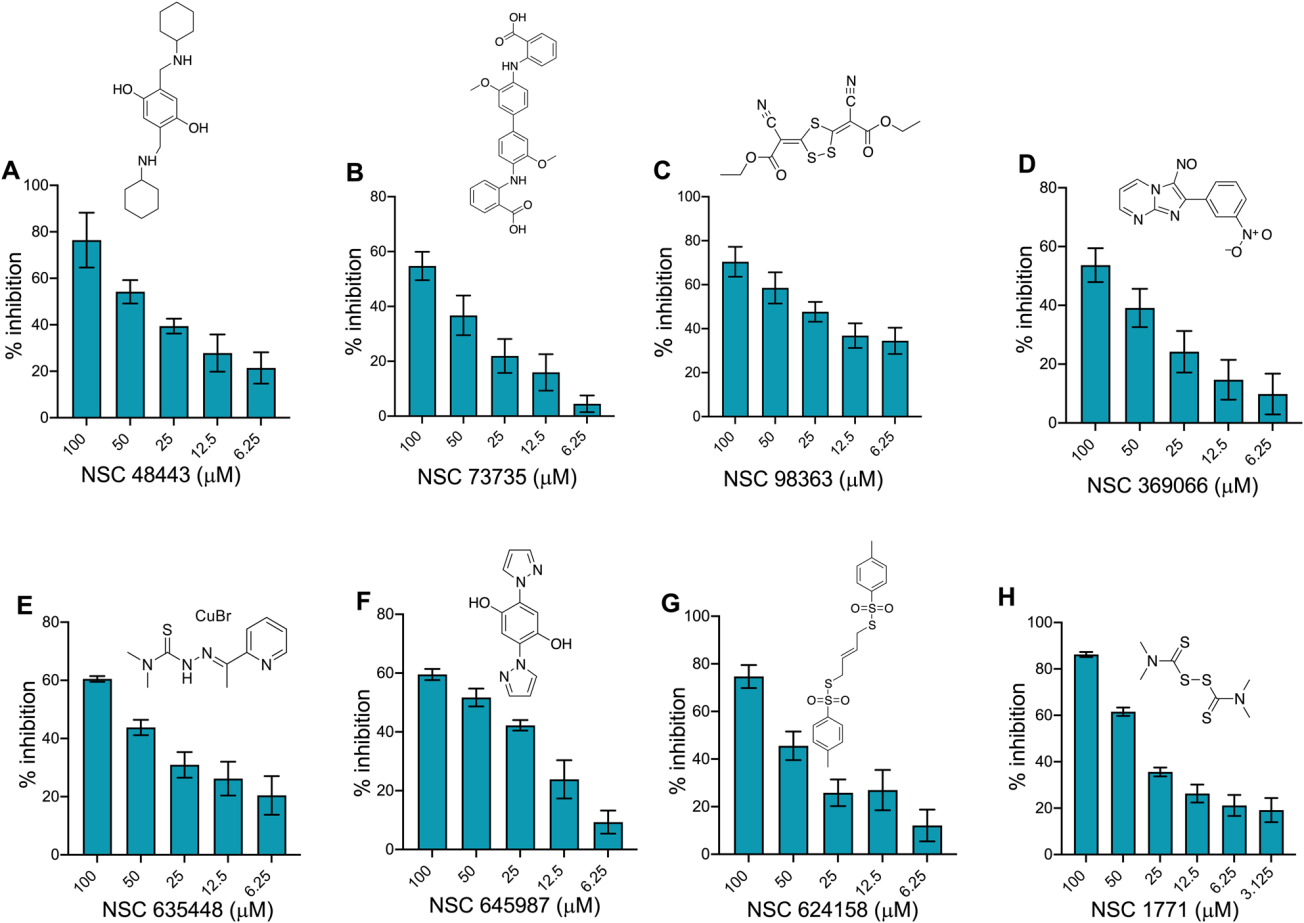
Dose response inhibition of Rv3341 activity by the identified primary hits. MetX enzymatic assays were performed in the presence of different concentrations of primary hits and percentage inhibition was calculated as described in Materials and Methods. The data shown in y-axis represents mean ± S.E. of percentage inhibition obtained from three independent experiments performed in duplicates. The structures of various primary hits are also shown in these panels.

We note that the identified small molecules were structurally different from known HSAT inhibitors. Therefore, we next performed inhibition assays in the presence of different concentration of the identified small molecules. As shown in Fig. 4, the identified primary hits inhibited enzymatic activity of *M. tuberculosis* MetX in a dose dependent manner. These observations suggests that the identified primary hits might possess broad spectrum anti-microbial activity as they are able to inhibit enzymatic activity associated with *M. tuberculosis* and *S. aureus* HSAT homologs. Taken together, our target-based screen identified various small molecules that were able to inhibit acetyl transferase activity associated with HSAT homologs from *M. tuberculosis* and *S. aureus*.

### Molecular docking and binding studies of the primary hits on *M. tuberculosis* and *S. aureus* HSAT proteins

The surface visualization of three dimensional *M. tuberculosis* and *S. aureus* HSAT structures in Discovery Studio revealed a small groove near the catalytic triad of both homologs (Fig. S2B–D). Thus, this groove of HSAT proteins was targeted for docking studies with small chemical inhibitors. The molecular docking experiments were performed for acetyl-CoA, L-homoserine and the identified small molecule inhibitors in the three dimensional structure of Rv3341 as described in Materials and Methods. We observed that the binding free energies for interaction of these small molecules with Rv3341 were in the range of − 6.0 to − 8.0 kcal/mol except for NSC1771. Similar trend in docking scores of primary hits were obtained in *S. aureus* HSAT (Table 1). The binding free energies of these primary hits in *S. aureus* HSAT protein were in the range of − 4.0 to − 8.0 (Table 1). Most of the docked small chemicals binding free energies with Rv3341 were comparable with the binding free energy for acetyl-CoA (-8.0 kcal/mol) and better than the binding free energy obtained for interaction of the other substrate, L-homoserine (− 4.4 kcal/mol). The binding free energies for interaction of acetyl-CoA and L-homoserine with *S. aureus* HSAT were -6.5 kcal/mol and − 4.3 kcal/mol, respectively, which is comparable with the binding energy of acetyl-CoA and L-homoserine with Rv3341.

**Table 1:** The details of interacting residues in docked poses of primary hits in Rv3341 modelled protein using AutoDock Vina.

CID No	NSC number	MIC_99_ value (µM)	Rotatable bonds	AutoDock Vina binding free energy (kcal/mol) (*M. tuberculosis* HSAT)	Interactions (MTB MetX)	AutoDock Vina binding free energy (kcal/mol) (*S. aureus* HSAT)	TC_50_ value (µM)
1	NSC 1771	1.56–3.125	5	− 4.1	–	− 3.9	25
2	NSC 98363	25	6	− 6.4	HB: Thr61, Gly62, Arg227	− 5.0	> 50
3	NSC 48443	200	8	− 7.9	Cation-π: Arg227, Arg276	− 7.8	> 50
4	NSC 635448	3.125	4	− 6.6	HB: Thr61	− 6.0	< 5
5	NSC 624158	200	8	− 8.0	HB: Arg227, Lys272, Arg276 Cation-π: Arg227, Lys272	− 7.9	> 50
6	NSC 73735	> 200	11	− 7.8	HB: Thr61, Arg227 Cation-π: Lys272, Arg276	− 4.3	> 50
7	NSC 369066	6.25	3	− 8.0	HB: Leu60, Met158, Arg227, Tyr234 Cation-π: Arg276	− 7.2	12.5
8	NSC 645987	25	4	− 7.3	HB: Arg227, Arg276	− 5.8	50
9	L-Homoserine	–	6	− 4.4	HB: Leu60	− 4.3	–
10	Acetyl-CoA	–	29	− 8.0	HB: Leu60, Gly78, Arg227, His231, Gln269, Arg276	− 6.5	–

HB stands for Hydrogen bond.

**Figure 5:**
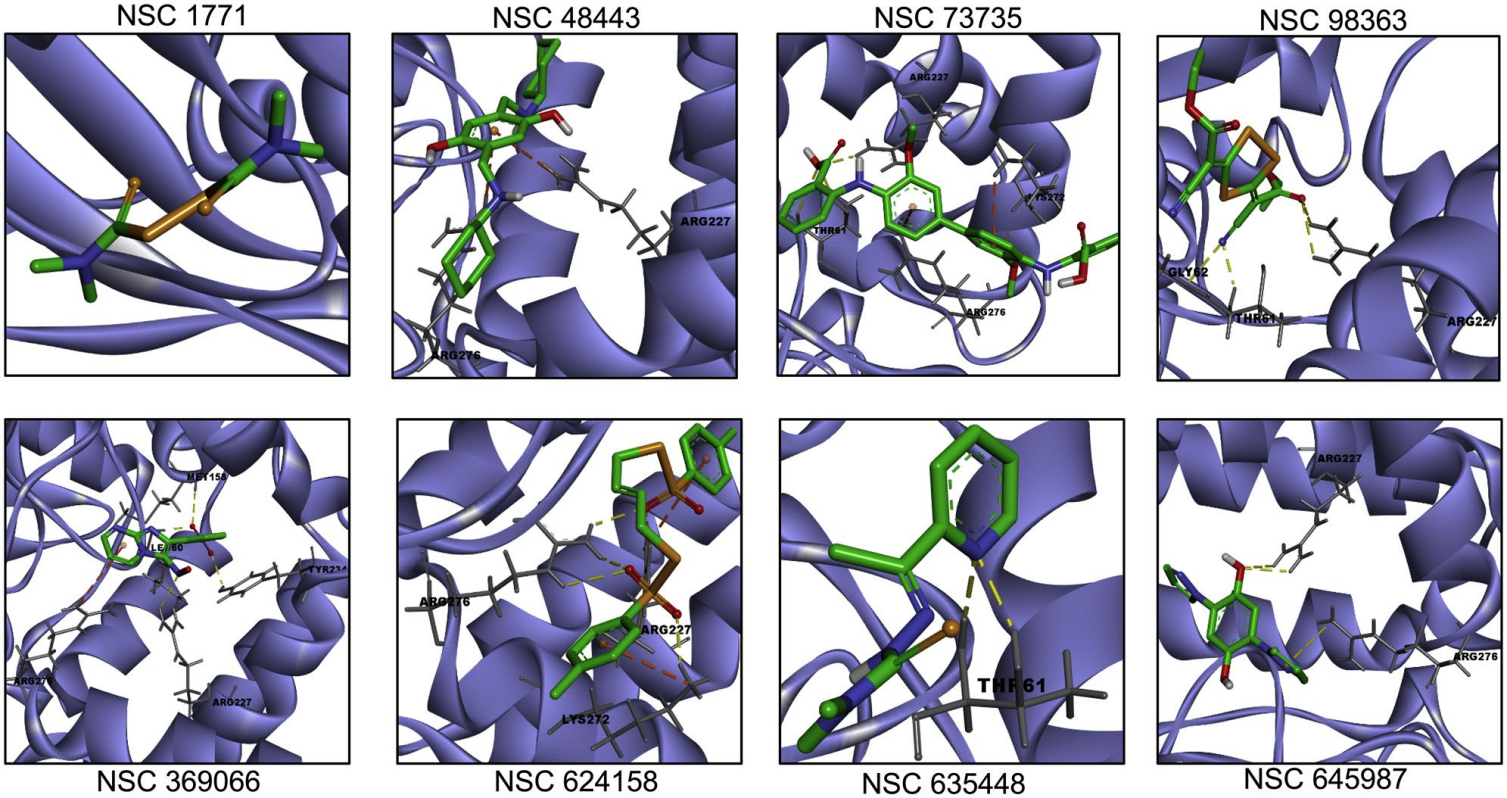
Molecular docking of the identified primary hits in MetX structure from *M. tuberculosis*. Docking of the identified primary hits in the three-dimensional structure of Rv3341 is shown. The hydrogen bonds and cation-pi bonds have been shown as yellow dotted and orange dotted lines, respectively. The residues involved in these interactions are also labeled in the panels.

**Figure 6:**
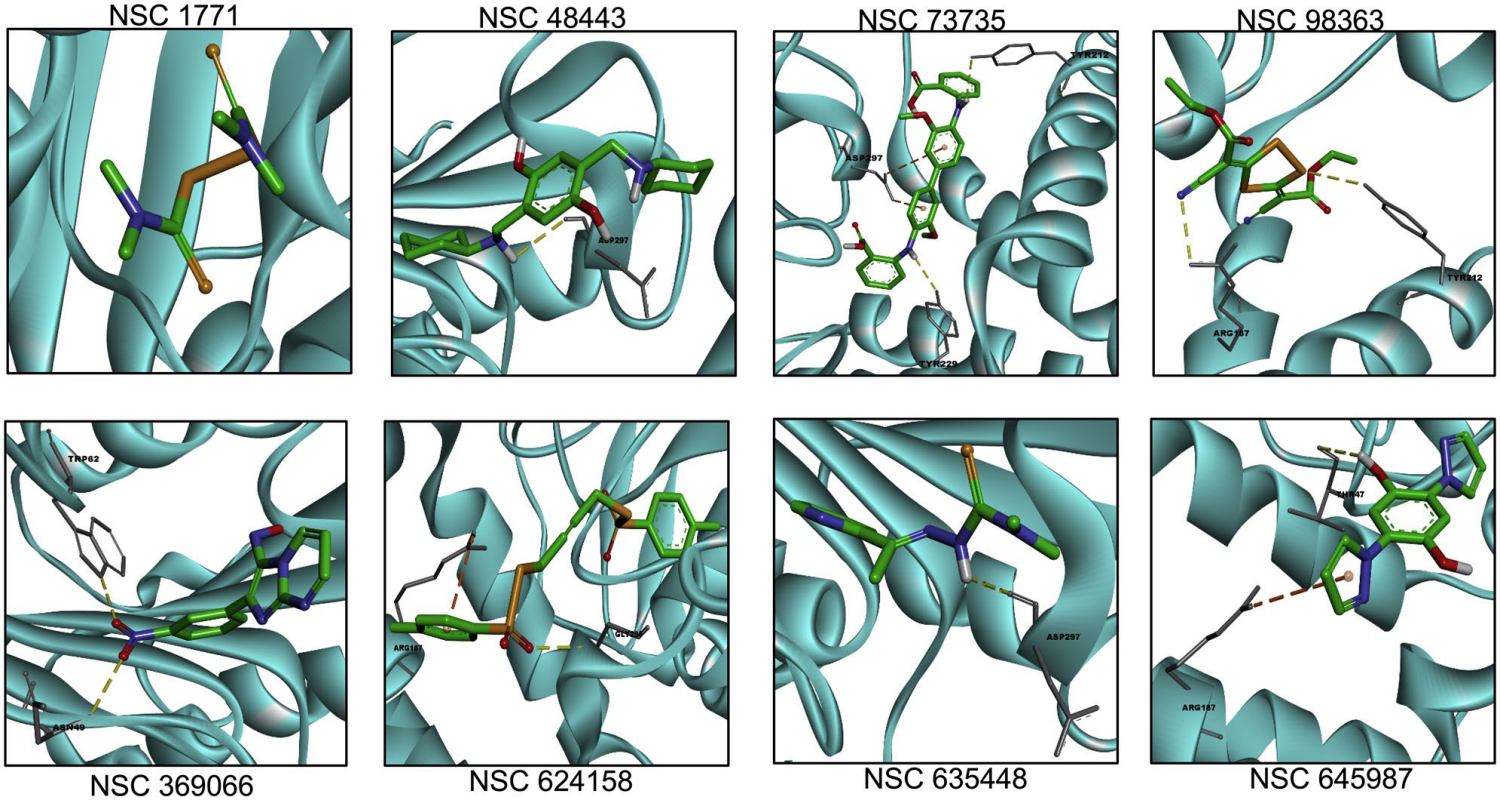
Molecular docking of the identified primary hits in HSAT structure from *S. aureus*. Docking of the identified primary hits in the three-dimensional structure of *S. aureus* HSAT protein is shown. The hydrogen bonds and cation-pi bonds have been shown as yellow dotted and orange dotted lines, respectively. The residues involved in these interactions are also labeled in the panels.

**Figure 7:**
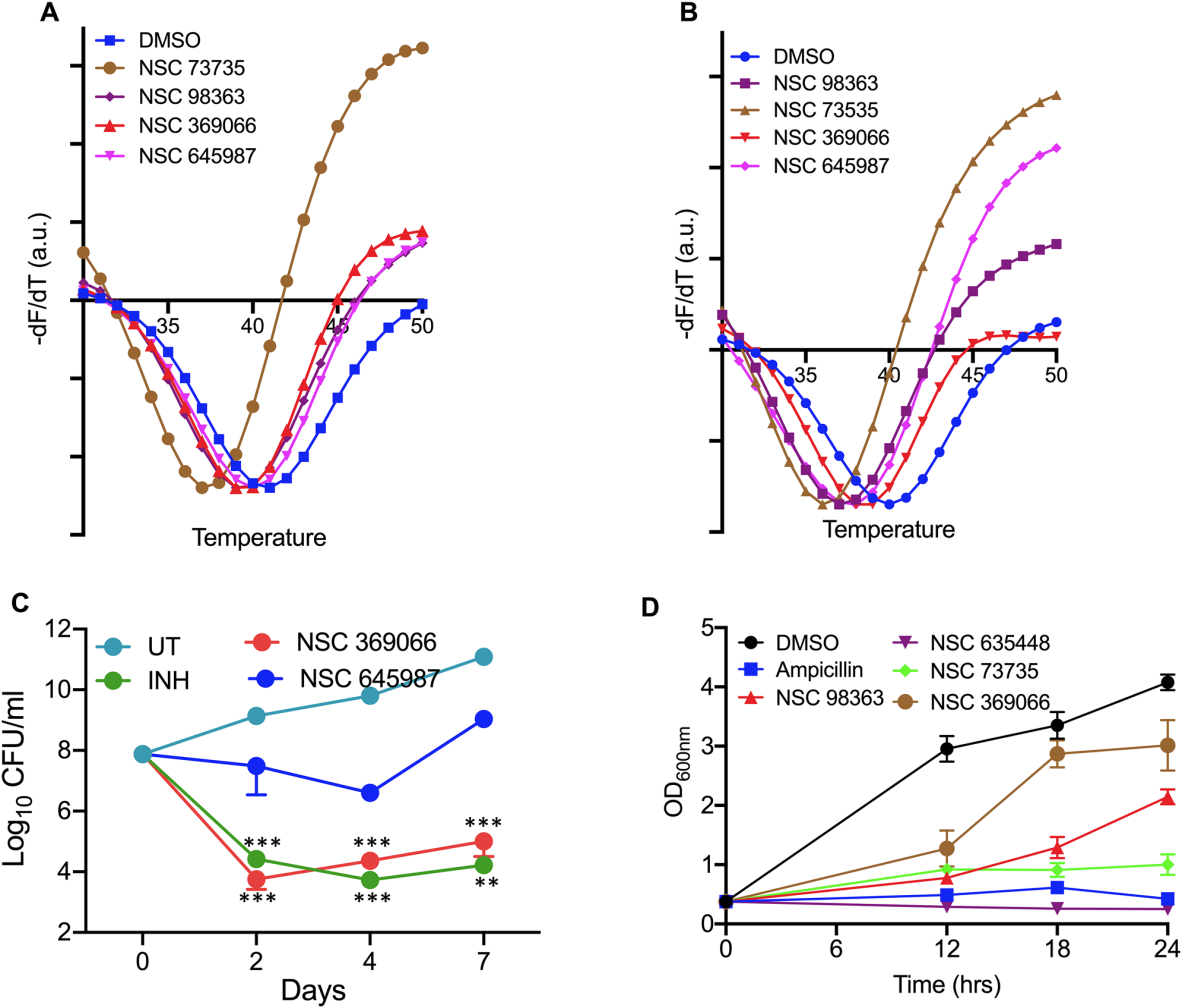
Primary hits bind HSAT homologs from *M. tuberculosis* and *S. aureus*. (**A**,**B**) ThermoFluor binding assays in the presence of primary hits was determined with HSAT homolog from *M. tuberculosis* (**A**) or *S. aureus* (**B**). The data shown in this panel is representative of three independent experiments (**C**) Time kill kinetics of NSC369066, NSC645987 and INH against *M. tuberculosis* H37Rv. Early-log phase cultures of *M. tuberculosis* H37Rv were exposed to 10 × MIC_99_ concentration of either NSC369066 or NSC645987 or INH and CFU numbers were determined at day 2,4 and 7 post-exposure. The data shown is mean ± S.E. of log_10_ CFU obtained from triplicates performed in duplicates. Statistical differences were observed for the indicated groups, One-way ANOVA using Dunnett method **P < 0.01, ***P < 0.001. (**D**) Growth assays of *S. aureus* in the presence of identified primary hits. Early-log phase cultures of *S. aureus* were exposed to 100 µM concentration of either NSC635448 or NSC73735 or NSC98363 or NSC369066 and OD_600nm_ was determined at regular intervals. The data shown in this panel is mean ± S.E. of data obtained from two independent experiments performed in duplicates.

The detailed analysis of the docked poses revealed among the primary hits, NSC 98363, NSC73735, and NSC635448 formed hydrogen bond with Thr61 of Rv3341 (Fig. 5, Table 1). Interestingly, Thr61 is highly conserved residue among various HSAT homologs and lies adjacent to the catalytic triad in Rv3341 three dimensional structure. In concordance, we also observed that acetyl-CoA is also involved in hydrogen bond formation with Thr47 of *S. aureus* HSAT protein (corresponding to Thr61 in Rv3341) in docked poses (Fig. S2F). Along with Thr61, another highly conserved residue, Leu60 is observed to form hydrogen bond with NSC369066 and both the substrates, L-homoserine and acetyl-CoA (Fig. 5, Fig. S2E). In addition to these, we also observed Arg227, Lys272 and Arg276 that were adjacent to catalytic tunnel also involved in hydrogen bond formation and/or cation-pi interaction with several primary hits (Fig. 5). The corresponding Arg187 residue in *S. aureus* HSAT homolog was also involved in hydrogen bond formation with acetyl-CoA (Fig. S2F). In our molecular docking experiments, we observed that NSC369066 and NSC624158 seemed to be the best fit in Rv3341 binding pocket (Fig. 5, Table 1). NSC1771 was the worst fit based on binding free energy and no notable bond formation was observed with either of the HSAT homologs (Figs. 5, 6 and Table 1). The binding of the primary hits with HSAT homolog from *M. tuberculosis* and *S. aureus* was determined by ThermoFluor assays. As shown in Fig. 7A,B, we observed that these small molecules had a destabilizing effect on HSAT homolog from both *M. tuberculosis* and *S. aureus*. In the case of Rv3341, we observed a negative shift in T_m_ of 1 °C, 2 °C, 2 °C and 4 °C in the presence of NSC645987, NSC98363, NSC369066 and NSC73735, respectively (Fig. 7A). In the case of *S. aureus* homolog, a negative shift in T_m_ of 2 °C, 3 °C, 1 °C, and 4 °C, in the presence of NSC645987, NSC98363, NSC369066 and NSC73735, respectively, was observed (Fig. 7B).

### In vitro killing activity of the primary hits

Next, we performed assays to evaluate the in vitro antitubercular activity of the identified MetX inhibitors. NSC635448 and NSC369066 were found to be the most potent compounds displaying MIC_99_ values of 3.125 µM and 6.25 µM against *M. tuberculosis*, respectively (Table 1). The MIC_99_ values of the remaining primary hits NSC98363, NSC48443, NSC624158, NSC73735 and NSC645987 was 25 µM, 200 µM, 200 µM, > 200 µM and 25 µM, respectively (Table 1). We also performed cell viability experiments to determine cytotoxicity of the identified primary hits against THP-1 macrophages. As shown in Table 1, NSC635448, the most potent hit identified from our screen was cytotoxic in THP-1 macrophages even at 5 µM concentration. The TC_50_ values of NSC369066 and NSC1771 against THP-1 macrophages was 12.5 µM and 25 µM, respectively. The remaining identified small molecules were not cytotoxic in THP-1 macrophages even at 50 µM concentration.

**Figure 8:**
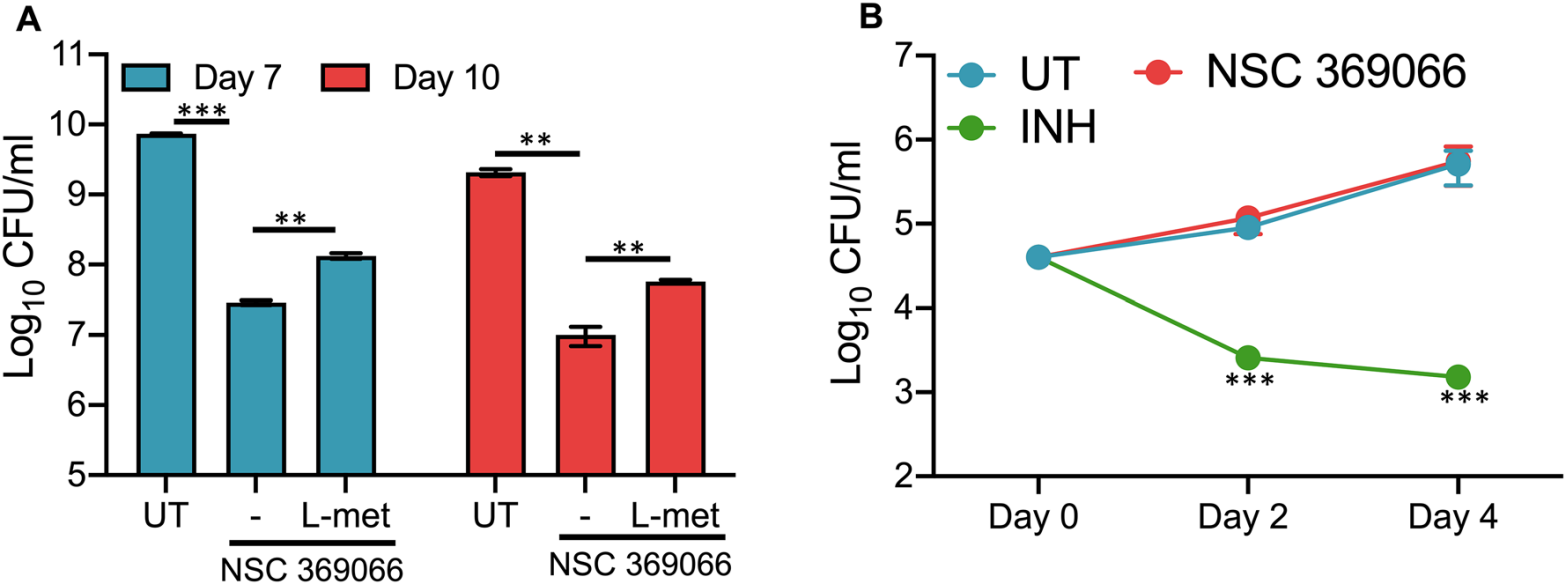
Activity of NSC369066 against *M. tuberculosis* in liquid cultures in the presence of L-methionine and in macrophages. (**A**) The cultures of *M. tuberculosis* were exposed to 5 × MIC_99_ NSC369066 in the presence or absence of 250 µg/ml L-methionine and bacterial numbers were determined at day 7 and 10 post-exposure. The data shown in this panel in mean ± S.E. of log_10_ CFU obtained from two experiments performed in duplicates. Statistical differences were observed for the indicated groups, paired (two-tailed) t-test, **P < 0.01, ***P < 0.001. (**B**) Intracellular *M. tuberculosis* growth at 48 h and 96 h after treatment of THP-1 macrophages with INH or NSC369066 at 10 µM and 12.5 µM, respectively. The data shown in this panel in mean ± S.E. of log_10_ CFU obtained from three independent experiments performed in duplicates. Statistical differences were observed for the indicated groups, One-way ANOVA using Dunnett method ***P < 0.001.

Next, we performed in vitro killing experiments after exposure of *M. tuberculosis*, H_37_Rv to NSC369066 and NSC645987 for 2, 4 and 7 days. Levels of killing noticed in NSC369066 treated samples was also comparable to INH-treated samples at 10 × MIC_99_ concentration (Fig. 7C). However, less killing was observed in NSC645987 treated samples (Fig. 7C). This might be attributed to the intracellular stability of the compound or emergence of resistance upon continuous exposure to the drug. Next, we performed experiments to determine the growth inhibition activity of the identified hits against *S. aureus*. As shown in Fig. 7D, growth inhibition of *S. aureus* was observed after exposure to either NSC635448 or NSC73735 or NSC98363. The growth inhibition observed in the presence of either NSC635448 or NSC73735 was similar to that observed in the presence of ampicillin (Fig. 7D). We did not observe any growth inhibition of *S. aureus* in the presence of NSC48443 and NSC645987 (data not shown). These observations suggested that NSC369066 binds to MetX and inhibits the in vitro growth of *M. tuberculosis*. In order to further validate our findings, we performed in vitro killing experiments of *M. tuberculosis* upon exposure to NSC369066 in liquid cultures upon supplementation with L-methionine. As shown in Fig. 8A, we observed significant killing in NSC369066 treated cultures after exposure for either 7 days or 10 days. However, we did not observe complete restoration upon supplementation of 7H9 medium with L-methionine. As shown in in Fig. 8A, supplementation with L-methionine partially restored NSC369066 mediated killing by approximately 5.0-fold after 7 days or 10 days of exposure. The observed partial growth restoration in the presence of L-methionine suggests the presence of other targets for NSC369066 in vitro.

We also performed intracellular killing kinetic experiments by seeding THP1 macrophages, infecting them with *M. tuberculosis* and treated infected macrophages with either NSC369066 or INH. As expected, we observed significant reduction in CFU at day 4 of INH treatment in comparison to untreated cells (Fig. 8B). We did not see any differences in the bacterial counts of NSC369066-treated, and untreated macrophages at both 2- and 4- days post-infection (Fig. 8B). This might be attributed to poor intracellular concentration of NSC369066 in infected macrophages. Overall, in the present study we (i) developed an assay system to identify small molecule inhibitors against HSAT proteins, (ii) identified small molecules against HSAT proteins and (iii) the identified HSAT’s inhibitor demonstrating activity against *M. tuberculosis* and *S. aureus* in liquid cultures.

## Discussion

The genome of *M. tuberculosis* encodes for all enzymes involved in L- methionine biosynthesis^58^. *M. tuberculosis* needs a functional methionine/SAM biosynthesis for survival in the host^31^, suggesting that the enzymes belonging to this pathway are attractive targets for designing new anti-tubercular therapy. Here, we have biochemically characterized and validated MetX enzyme from *M. tuberculosis* as a drug-target. Multiple sequence alignment studies revealed that percentage identity among HSAT homologs from various microorganisms is between 20 and 30%. Using CRISPRi knock down approach, we determined the in vitro essentiality of Rv3341 in liquid cultures. We observed a slower growth rate of the knock down strain in comparison to the parental strain. qPCR studies revealed that the transcript levels of *metX* were reduced by ~ 15.0–20.0-fold in knock down strains. These observations suggests that the residual amount of MetX protein in knock down strain was sufficient for growth of *M. tuberculosis* in liquid cultures. However, in a previous study it has been reported that the *metX* deletion mutant strain of *M. tuberculosis* was unable to grow in vitro in liquid cultures in the absence of L-methionine^31^. In the same study the authors showed that *metX* deletion mutant strain was unable to grow in immunocompromised and immunocompetent mice^31^. These observations suggests that *M. tuberculosis* is unable to scavenge L-methionine from host tissues to promote its intracellular growth. The amount of L-methionine in human and mice serum is ~ 4–5 µg/mL and 8 µg/mL, respectively^59,60^. Berney et al., also demonstrated that supplementation of liquid medium with 3 µg/mL of L-methionine was able to restore the growth defect associated with the *metX* deletion mutant strain in liquid cultures. In agreement, inhibition of L-methionine biosynthesis also results in growth arrest and attenuation of virulence in fungi^61^. These observations indicates that blocking synthesis of L-methionine and S-adenosyl methionine results in rapid death and HSAT is a target worth exploring for development of effective anti-microbial compounds.

Here, we have performed high throughput screening to identify inhibitors against HSAT homolog from *M. tuberculosis*. HSAT enzymes from various microorganisms exists as monomer or dimers, however, we observed that Rv3341 exists as a monomer or dimer or tetramer in solution^53,54,62,63^. In our assays, the three different forms of (His)_6_-Rv3341 displayed comparable activity in vitro*.* We observed that MetX enzyme followed Michaelis–Menten kinetics with a *k*_*cat*_*/K*_*m*_ of 0.134 µM^−1^ min^−1^for acetyl-CoA. In concordance with *H. influenzae* homolog, (His)_6_-MetX was also able to use propionyl-CoA as an acyl donor, however, no activity was seen in the presence of succinyl-CoA^64^. In our enzymatic assays, we observed that there is no dependence of HSAT activity on pH of the assay buffer and the maximal activity was attained in the initial 10 mins of the enzymatic reaction. The activity of (His)_6_-MetX was also unaltered upon the addition of metal ions or TritonX-100 in the assay buffer.

The structure of HSAT shares an overall topology of the α/β hydrolase family superfamily as reported in the case of *Haemophilus influenzae*, *Leptospira interrogans* and *S. aureus*^45,53,54^. The catalytic triad comprising of a serine, histidine and aspartic acid residue is the most conserved feature of α/β hydrolase family and the enzymatic reaction catalysed by HSAT homologs follow ping pong mechanism^54^. In the first reaction, the acyl-group is transferred to a nucleophile resulting in the formation of acyl-enzyme intermediate and subsequently the acyl group is transferred to L-homoserine^64,65^. The catalytic triad residues are also present in Rv3341 and serine at 157 position is essential for its acetyl-transferase activity. Since, we did not observe significant differences in the Far-UV CD spectra for parental and Rv3341^S157A^, we inferred that this residue is vital for the activity of MetX protein. We have screened a small molecule library of 2443 compounds in 96-well format using CoA release assay and identified 8 compounds that inhibited HSAT activity in the range of 60% to 80%. The identified compounds were structurally different from known fungal-HSAT inhibitors, CTCQC, a nucleotide substrate analog and Ebelactone A^66,67^. However, in another study, it has been shown that Ebelactone A, is unable to inhibit the activity associated with *S. aureus* homolog. The authors concluded that these differences observed in the inhibition assays between HSAT homologs is most likely due to the narrower catalytic tunnel in *S. aureus*^45,66^. However, the hits identified in the present study were also able to inhibit activity associated with *S. aureus* homolog, thereby suggesting that these small molecules might possess broad-spectrum antimicrobial properties. All initial hits except NSC635448 and NSC369066 displayed anti-tubercular activity of greater than 10 µM in whole-cell based assay, which could be attributed to their ineffective transport and/or intracellular metabolism. NSC635448 was the most potent small molecule against both *M. tuberculosis* and *S. aureus*. However, NSC369066 displayed MIC_99_ value of 6.25 µM against *M. tuberculosis* but was inactive in our *S. aureus* growth inhibition assays. The exposure of *M. tuberculosis* to NSC369066 inhibited its growth in a bactericidal manner and this was partially restored upon supplementation of medium with L-methionine. Similar supplementation studies have been performed to validate enzymes of other amino acid biosynthetic pathways from *M. tuberculosis* as drug-targets^31,35,68–70^. Although, NSC73735 was inactive against *M. tuberculosis*, it showed good activity against *S. aureus* in liquid cultures. The variations observed for in vitro killing of *S. aureus* or *M. tuberculosis* by the identified primary hits might be attributed to the differences in the intracellular drug levels attained in *S. aureus* or *M. tuberculosis*. Various factors such as uptake or efflux along with intracellular stability of the drugs might contribute to this phenomenon.

Molecular docking studies also provided additional details about the mechanism of inhibition of Rv3341 by the identified small molecules. We observed that except NSC73735, the free binding energy values for the remaining primary hits was similar or better than those observed in the case of binding of acetyl-CoA and L-homoserine. Also, docking studies revealed that either Thr 61 or Arg 227 or both residues of *M. tuberculosis* MetX are involved in hydrogen bond formation with the identified primary hits. The corresponding residues in *S. aureus* were also involved in interaction with the identified small molecules. ThermoFluor binding assays resulted in a temperature shift of 1–4 °C, thus confirming, conformational changes in HSAT homolog from *M. tuberculosis* and *S. aureus* as a result of small molecule binding. In our screen, NSC1771 (Thiram) was a false hit identified as no notable bond formation was observed upon molecular docking of Thiram with either HSAT homolog from *M. tuberculosis* or *S. aureus*. We speculate that false selection of Thiram as a MetX inhibitor might be attributed to its property of forming disulfides with thiol-bearing molecules such as Coenzyme A, a major product of the enzymatic reaction^71^. The observed antimycobacterial activity of Thiram in vitro might be associated with alteration of copper homeostasis as reported in the case of Disulfiram, an organo-sulfur based, FDA approved drug used for the treatment of chronic alcholism^72,73^. NSC73735, redoxal has also been reported to inhibit decaprenyl diphosphate synthase (DPPS) of *M.tuberculosis,* an enzyme involved in cell wall biosynthesis^74^*.* In concordance with our results redoxal has been shown to be inhibit *S. aureus* replication in *C. elegans* infection model^74^*.* Besides this enzyme, redoxal has also been shown to inhibit dihydroorotate dehydrogenase and ribonucleotide reductase enzyme^75,76^. Further, redoxal has also been shown to inhibit replication of influenzae A and HIV^77,78^. In concordance with our results, NSC98363 has also been shown to inhibit growth of *S. aureus* by targeting prenyl transferase enzyme^74^. NSC635448, was also identified in other high throughput screening assay to inhibit growth of ovarian cancer stem-like cells^79^. NSC635448 has also been shown to be active against phenotypically drug-tolerant *M. tuberculosis* and also inhibits reverse transcriptase enzyme from HIV^80,81^. NSC624158, another small molecule identified from our screen has been shown to inhibit nicotinamide mononucleotide adenylyl transferase (NMNAT), an enzyme involved in NAD biosynthesis^82^. Taken together, this is the first study that has resulted in the identification of small molecule molecules that inhibit the acetyl transferase activity of HSAT from *M. tuberculosis* and *S. aureus*.

A major concern in the field of chemotherapy that needs to be addressed is the lengthy duration of treatment for individuals with drug-resistant TB. According to WHO reports, the number of resistant cases in India are dreadful and the number of MDR-TB, XDR-TB and TDR-TB cases are going to worsen further in 2025. In addition to TB, the incident rates for other bacterial pathogens are also on the rise due to the emergence of drug-resistant strains. The disease caused by *S. aureus* is resistant to available drugs by ~ 64% and are classified as MRSA infection. Therefore, the pace of validating new drug targets and identifying new drugs should be fast enough to counter the issue of antimicrobial resistance. Methionine biosynthesis pathway is (i) involved in several biological processes such as translation initiation, synthesis of SAM, DNA and sulphur containing compounds, (ii) absent in mammalian cells, and (iii) predicted to be essential for the growth of microorganisms^31,66^. Our findings suggest that exposure to drugs targeting these biosynthetic pathway might lead to a metabolic shutdown in *M. tuberculosis* and *S. aureus*. To the best of our knowledge, this is first study, where high throughput screening has been performed to identify small molecule inhibitors targeting HSAT enzyme from *M. tuberculosis*. NSC369066 was identified as the most potent compound from our screen that also possessed whole cell activity against *M. tuberculosis*. In the presence of L-methionine, we observed a partial restoration of NSC369066 mediated *M. tuberculosis* killing.

These findings imply that NSC369066 has a multi-target killing mechanism and inhibits *M. tuberculosis* growth by targeting various enzymes, including MetX, and represent a limitation of the current study. However, NSC369066 showed no activity in our ex vivo experiments.

The lack of a three-dimensional structure for MetX in the complex with NSC369066 is another limitation of the study, despite the fact that the protein-small molecule interactions was validated by Thermo Fluor assays. Therefore, future experiments would focus on finding small molecule inhibitors that have improved binding affinity with MetX and activity against both intracellular drug-susceptible and drug-resistant bacteria. The optimised leads would be validated in various biochemical, structural biology, L-methionine supplementation and MIC_99_ determination assays using *metX* knock down strain. Since HSAT is a novel unexplored drug-target, we anticipate that the identified small molecules would possess broad-spectrum anti-microbial activity against both drug-sensitive and drug-resistant bacteria.

## Supplementary Information

Supplementary Information.

## Data Availability

Rv3341 gene sequence was obtained from Uniprot database (Accession number P9WJY9). The complete data generated and analysed in the current study is available in the manuscript.
