# Supplementary material for: Identification of small molecules targeting homoserine acetyl transferase from Mycobacterium tuberculosis and Staphylococcus aureus
Source: Sci Rep. 2022 Aug 13;12:13801. doi: 10.1038/s41598-022-16468-w (PMC9376091; doi:10.1038/s41598-022-16468-w)
Supplement: Supplementary file 1 — Supplementary Information. [file 41598_2022_16468_MOESM1_ESM.pdf]

Identification of small molecules targeting homoserine acetyl transferase from *Mycobacterium tuberculosis* and *Staphylococcus aureus*

Deepika Chaudhary[1,2], Avantika Singh[3], Mardiana Marzuki[4], Abhirupa Ghosh[5], Saqib Kidwai[1], Tannu Priya Gosain[1], Kiran Chawla[2], Sonu Kumar Gupta[1], Nisheeth Agarwal[1], Sudipto Saha[5], Yashwant Kumar[1], Krishan Gopal Thakur[3], Amit Singhal[4,6,7] and Ramandeep Singh[1#]

[1] Translational Health Science and Technology Institute, NCR Biotech Science Cluster, Faridabad, Haryana- 121001, India.
[2] Manipal academy of higher education, Manipal, Karnataka – 576104. India.
[3] Structural Biology Laboratory, Council of Scientific and Industrial Research-Institute of Microbial Technology , Chandigarh-160036, India.
[4] Infectious Diseases Labs (ID Labs), Agency for Science, Technology and Research (A*STAR), Singapore 138648.
[5] Division of Bioinformatics, Bose Institute, Kolkata, West Bengal 700054, India.
[6] Singapore Immunology Network (SIgN), (A*STAR), Singapore 138648.
[7] Lee Kong Chian School of Medicine, Nanyang Technological University, Singapore 308232, Singapore.

[#]Corresponding author Mailing address: Tuberculosis Research Laboratory, NCR Biotech Science Cluster, 3rd Milestone, Faridabad-Gurugram Expressway. PO Box # 4. Faridabad – 121001. Email: ramandeep@thsti.res.in

Running Title: Targeting HSAT enzyme from *M. tuberculosis and S. aureus.*

Keywords: *Mycobacterium tuberculosis*, target based screening, homoserine acetyl transferase, small molecule inhibitors, methionine biosynthesis.

Supplementary figures legends.

Figure S1: Multiple sequence alignment among HSAT proteins. Multiple sequence alignment among HSAT proteins from various microorganisms was performed using Clustal Omega software and viewed using GeneDoc. The highly conserved residues among HSAT homologs from various bacterial species are shaded in black.

Figure S2: (A) The structures of *S. aureus* HSAT., PDB id: 4QLO (Cyan) and *M. tuberculosis* HSAT, PDB id: 6PUX (violet) were aligned and visualized using Pymol. **(B)** The surface around the *M. tuberculosis* Rv3341 with the catalytic triad and 3 other residues shown in red and visualized in Discovery Studio. **(C)** A small groove marked with black circle is observed near the catalytic triad of Rv3341. **(D)** A small groove marked with black circle is observed near the catalytic triad of *S. aureus* HSAT. (**E and F)** Molecular docking of acetyl-CoA and L-homoserine in the three dimensional structure of Rv3341 (E) and *S. aureus* (F) was performed as described in Materials and Methods. The H-bonds are shown as yellow dotted lines and cation-pi bonds are shown as orange dotted lines in this panel. The residues involved in these reactions are labeled in black.

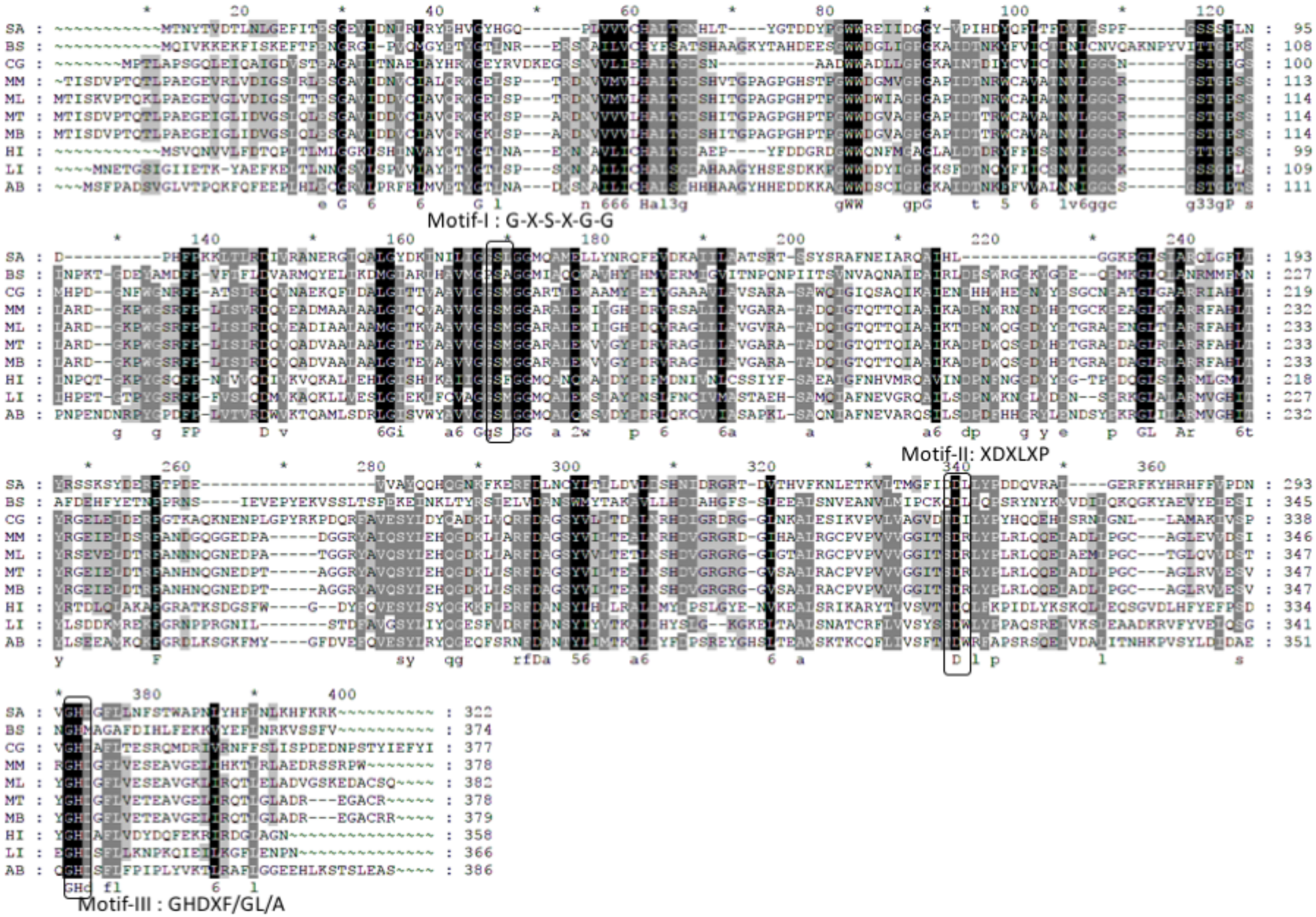

Chaudhary et al., Figure S1

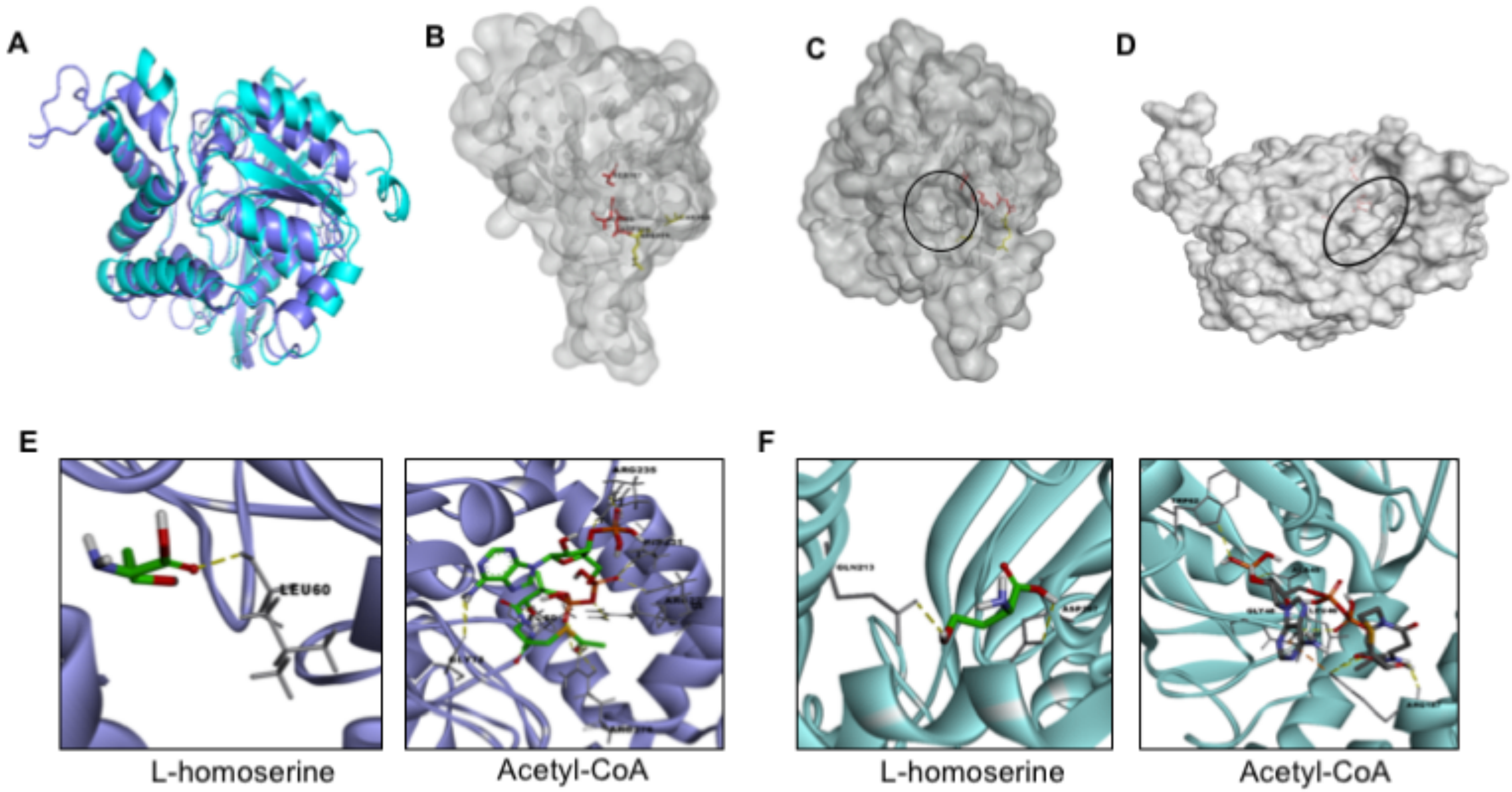

Chaudhary et al., Figure S2

Table S1- List of Bacterial strains, cell lines, plasmids and primers used in the study.

**Bacterial strains**		
XL-1 blue	*recA1*, *endA1 gyrA*96 thi-1 *hsdR17 supE44 relA1 lac [F'proAB lac IqZ∆*M15 Tn10(Tet^R)]	Stratagene, USA
BL-21 *plysS*	*F-, ompT, hsdS$_B$(r$_b$-m$_b$), dcm, gal, λ(DE3), plysS, Cm^R*	Promega, USA
M. tuberculosis H37Rv	*M. tuberculosis* parental strain	ATCC27294
M. tuberculosis -pTetInt-dCas9	*M. tuberculosis* virulent strain harboring pTetInt-dCas9	Chaudhary et al., 2015
M. tuberculosis - gRNA	*M. tuberculosis* strain harboring pTetInt-dCas9 and *pGrna*	This study
M. tuberculosis - gRNA-*metX*	*M. tuberculosis* strain harboring pTetInt-dCas9 and *pGrna-metX*	This study
Staphylococcus aureus	*Staphylococcus aureus* parental strain	ATCC25923, a kind gift from Dr. Bhabatosh Das.
THP1	Human monocyte cell line	NCCS PUNE
**Plasmids**		
pGEM-T Easy	TA cloning vector, *amp^r*	Promega, USA
pGEM-T Easy-Rv3341	pGEM-T Easy harboring Rv3341	This study
pGEM-T Easy - Rv3341 ^{S157A}	pGEM-T Easy harboring Rv3341^{S157A} mutant	This study
pET28b	T7 based prokaryotic expression system	Novagen, UK
pET28b-Rv3341	pET28b harboring Rv3341	This study
pET28b-Rv3341^{S157A}	pET28b harboring Rv3341^{S157A}	This study
pET28b-HSAT^{SA}	pET28b harboring HSAT homolog from *S. aureus*	This study
pGrna	Anhydrotetracycline based expression system to express guide RNA	Chaudhary et al., 2015
pGrna-metX	pGrna harboring *metX* specific guide RNA.	This study
**Primers**		
	Forward (5'---3')	Reverse (5'------3')
Rv3341-ORF	GCATATGACGATCTCCGATGTACCCACCC	GAAGCTTTCACCGCCGACACGCGCCTTCACGA
HSAT^{SA}-ORF	GGGATCCGATGACAAATTACACAGTAGATACTT	GAAGCTTTTACTTACGCTTAAAATGCTTTAAATT
Rv3341^{S157A}	CGTCGGCGGCGCCATGGGCGGCG	CGCCGCCCATGGCGCCGCCGACG
Rv3341- *SYBR*	GGCAGAGCGGCGACTACCACGAG	GCACCGCGTAGCGCCCGCCGGCCG
sigA- SYBR	CTGTACGCCACGCAGCTGATGACCG	GCCCCAGGTTGCCTTCCTGGATCAG
Rv3341-gRNA	GCGGGCAAGCGAGCTGGGC	CGGCCCAGCTCGCTTGCCCGCCATG

Table S2: Pecentage inhibition of MetA enzymatic activity obtained in the presence of 100 mM of small compounds belonging to either Diversity Set or Mechanistic Set

Diversity Set

Plate ID:Well ID	NSC NUMBER	% INHIBITION
4803:A2	32065	-3.18
4803:B2	1390	-2.71
4803:C2	19893	-0.94
4803:D2	752	0.94
4803:E2	755	-1.65
4803:F2	762	4.24
4803:G2	6396	-1.29
4803:H2	18509	5.65
4803:A3	362856	11.76
4803:B3	750	19.76
4803:C3	13875	17.88
4803:D3	27640	29.65
4803:E3	45923	-1.47
4803:F3	79037	16.00
4803:G3	102816	5.65
4803:H3	127716	6.59
4803:A4	409962	14.59
4803:B4	26271	22.59
4803:C4	34462	22.12
4803:D4	63878	28.71
4803:E4	66847	12.71
4803:F4	77213	14.59
4803:G4	85998	10.82
4803:H4	105014	8.47
4803:A5	109724	10.82
4803:B5	119875	24.94
4803:C5	122758	15.06
4803:D5	169780	16.00
4803:E5	218321	-0.47
4803:F5	686673	1.88
4803:G5	701852	-8.94
4803:H5	713563	-4.24
4803:A6	719344	13.65
4803:B6	719345	3.29
4803:C6	747972	6.12
4803:D6	775351	-6.59
4803:E6	3088	2.82
4803:F6	26980	-4.24
4803:G6	38721	-7.88
4803:H6	606869	-8.47
4803:A7	25154	-1.88
4803:B7	71423	10.82
4803:C7	138783	24.94

4803:D7	312887	19.29
4803:E7	681239	7.06
4803:F7	712807	5.65
4803:G7	719627	-9.88
4803:H7	750690	5.65
4803:A8	757441	13.18
4803:B8	279836	-9.41
4803:C8	698037	4.24
4803:D8	715055	17.88
4803:E8	755986	1.88
4803:F8	756645	5.65
4803:G8	740	-10.82
4803:H8	609699	8.24
4803:A9	732517	-0.94
4803:B9	737754	10.82
4803:C9	743414	10.35
4803:D9	747971	22.59
4803:E9	747974	9.41
4803:F9	750691	16.00
4803:G9	754230	-2.82
4803:H9	755605	7.06
4803:A10	760766	0.47
4803:B10	761431	18.35
4803:C10	763932	17.41
4803:D10	256439	19.29
4803:E10	747599	-3.29
4803:F10	747973	11.29
4803:G10	754143	-3.76
4803:H10	758253	-8.00
4803:H4	758487	6.47
4803:H5	761068	-2.82
4803:H6	765694	14.12
4803:H7	82151	16.94
4803:H8	123127	-1.41
4803:H9	141540	15.53
4803:H10	180973	4.12
4803:H11	256942	-8.00
4804:A2	702294	3.27
4804:B2	745750	1.96
4804:C2	616348	8.29
4804:D2	719276	5.43
4804:E2	758246	-0.75
4804:F2	764134	0.75
4804:G2	122819	-1.76
4804:H2	246131	8.84
4804:A3	758252	-5.28
4804:B3	628503	-8.29
4804:C3	761432	-2.26

4804:D3	125973	5.28
4804:E3	49842	-2.26
4804:F3	67574	2.76
4804:G3	226080	-1.76
4804:H3	733504	-9.30
4804:A4	3053	-6.28
4804:B4	24559	1.41
4804:C4	125066	-8.29
4804:D4	608210	9.80
4804:E4	683864	-8.79
4804:F4	613327	2.76
4804:G4	369100	-2.26
4804:H4	761388	-6.28
4804:A5	45388	-5.28
4804:B5	92859	2.26
4804:C5	9706	9.30
4804:D5	241240	7.29
4804:E5	718781	-0.25
4804:F5	296961	-0.25
4804:G5	721517	-5.78
4804:H5	266046	-9.30
4804:A6	8806	0.75
4804:B6	749226	-5.28
4805:A2	18473	-9.94
4805:B2	30041	-5.97
4805:C2	38968	0.80
4805:D2	42633	-5.97
4805:E2	48231	2.27
4805:F2	65248	-6.25
4805:G2	75071	-7.67
4805:H2	113532	-5.97
4805:A3	122276	0.85
4805:B3	123797	-9.94
4805:C3	135857	-5.68
4805:D3	148304	1.36
4805:E3	149877	1.08
4805:F3	173969	-8.51
4805:G3	200686	-4.55
4805:H3	679449	-7.95
4805:A4	4135	-3.13
4805:B4	4426	-4.83
4805:C4	4936	-5.40
4805:D4	5784	-8.81
4805:E4	7606	1.08
4805:F4	7950	-1.36
4805:G4	9441	-8.52
4805:H4	9489	-1.70
4805:A5	10427	0.28

4805:B5	10772	-3.98
4805:C5	11128	-0.28
4805:D5	11141	-5.68
4805:E5	12588	1.70
4805:F5	13213	-1.80
4805:G5	14767	-8.81
4805:H5	14771	-2.27
4805:A6	15133	-5.40
4805:B6	15776	-7.39
4805:C6	16021	-7.10
4805:D6	16162	-8.24
4805:E6	16631	-7.05
4805:F6	16646	-7.61
4805:G6	16873	-9.66
4805:H6	17796	-8.98
4805:A7	18415	-7.39
4805:B7	19219	3.35
4805:C7	19848	-6.25
4805:D7	20559	-7.67
4805:E7	20586	-0.85
4805:F7	22847	0.28
4805:G7	23225	-7.10
4805:H7	27389	0.57
4805:A8	27626	-3.98
4805:B8	28011	-1.99
4805:C8	28837	-8.81
4805:D8	29193	-2.50
4805:E8	29629	-5.91
4805:F8	29851	-4.77
4805:G8	29874	-8.52
4805:H8	31712	-5.34
4805:A9	34012	-1.99
4805:B9	34210	-3.98
4805:C9	34794	-5.68
4805:D9	34983	-3.69
4805:E9	35534	-0.57
4805:F9	35679	0.28
4805:G9	37408	-6.25
4805:H9	37883	2.56
4805:A10	40840	-1.70
4805:B10	41331	-1.99
4805:C10	42021	-1.99
4805:D10	42028	-4.83
4805:E10	42231	-5.40
4805:F10	42774	-3.69
4805:G10	43013	2.78
4805:H10	43512	-2.84
4805:A11	43546	9.09

4805:B11	43805	-9.94
4805:C11	11470	-0.57
4805:D11	54260	-0.28
4805:E11	51787	1.70
4805:F11	47881	3.69
4805:G11	41833	-1.14
4805:H11	40817	15.91
4806:A2	44680	-2.59
4806:B2	44819	-10.44
4806:C2	45117	-1.68
4806:D2	45641	-1.37
4806:E2	45719	-9.50
4806:F2	46273	-4.83
4806:G2	47496	-3.27
4806:H2	48422	-6.70
4806:A3	49252	-0.78
4806:B3	50751	1.40
4806:C3	50858	-2.02
4806:D3	51093	-9.68
4806:E3	53506	-2.34
4806:F3	54834	-7.32
4806:G3	55459	2.02
4806:H3	55573	-6.07
4806:A4	55957	-2.96
4806:B4	56914	-8.57
4806:C4	57741	-8.57
4806:D4	57890	-2.62
4806:E4	60239	-8.26
4806:F4	60373	-3.58
4806:G4	60377	-5.14
4806:H4	60530	-7.63
4806:A5	60548	-6.07
4806:B5	62129	-1.40
4806:C5	62318	2.96
4806:D5	62511	-0.47
4806:E5	62609	2.02
4806:F5	62840	-2.34
4806:G5	63311	-2.34
4806:H5	63314	-7.63
4806:A6	64952	-7.63
4806:B6	66241	-2.02
4806:C6	67307	-1.71
4806:D6	68657	11.06
4806:E6	70717	3.58
4806:F6	72292	-0.47
4806:G6	73482	-3.27
4806:H6	75585	5.14
4806:A7	75786	1.09

4806:B7	75846	-2.02
4806:C7	77422	4.52
4806:D7	77913	3.58
4806:E7	78609	0.16
4806:F7	78999	-1.09
4806:G7	79010	3.89
4806:H7	79582	-3.89
4806:A8	80807	-7.94
4806:B8	81462	2.34
4806:C8	82339	-3.27
4806:D8	82769	3.89
4806:E8	83076	3.27
4806:F8	83237	3.89
4806:G8	83339	-0.47
4806:H8	83345	-8.26
4806:A9	84200	-8.26
4806:B9	85331	-5.14
4806:C9	88882	-1.40
4806:D9	89720	-0.78
4806:E9	91438	-6.70
4806:F9	92264	-0.47
4806:G9	92753	-6.70
4806:H9	93260	-8.88
4806:A10	96979	-7.32
4806:B10	97090	-6.70
4806:C10	97104	-4.83
4806:D10	97538	5.14
4806:E10	99756	-3.31
4806:F10	100729	4.21
4806:G10	102025	-2.02
4806:H10	102509	-8.57
4806:A11	106045	-6.70
4806:B11	106261	-4.83
4806:C11	108655	-0.78
4806:D11	109176	-3.89
4806:E11	109528	1.40
4806:F11	109813	0.47
4806:G11	109885	-2.02
4806:H11	104969	0.47
4807:A2	109231	2.89
4807:B2	111107	4.13
4807:C2	112975	4.55
4807:D2	116565	8.68
4807:E2	117386	7.02
4807:F2	118832	14.05
4807:G2	119969	6.20
4807:H2	120312	0.41
4807:A3	122131	0.00

4807:B3	123458	0.00
4807:C3	125197	0.83
4807:D3	127216	4.96
4807:E3	127458	6.20
4807:F3	127947	8.26
4807:G3	128068	5.37
4807:H3	131982	4.55
4807:A4	134577	5.79
4807:B4	134580	5.37
4807:C4	134784	-0.41
4807:D4	134785	8.26
4807:E4	135351	7.85
4807:F4	136065	10.74
4807:G4	145180	5.79
4807:H4	147829	5.37
4807:A5	150982	7.27
4807:B5	151901	5.37
4807:C5	152632	3.72
4807:D5	154316	7.85
4807:E5	154718	10.33
4807:F5	155196	8.26
4807:G5	155698	4.55
4807:H5	155703	2.48
4807:A6	156571	10.33
4807:B6	160005	8.68
4807:C6	162915	1.24
4807:D6	163104	13.22
4807:E6	163158	10.33
4807:F6	163920	10.74
4807:G6	164965	4.96
4807:H6	166900	4.13
4807:A7	169458	9.92
4807:B7	169566	3.72
4807:C7	173101	9.50
4807:D7	173103	9.92
4807:E7	176324	10.74
4807:F7	177952	12.81
4807:G7	191029	7.85
4807:H7	194242	2.48
4807:A8	194243	14.88
4807:B8	195031	19.01
4807:C8	203065	15.70
4807:D8	206630	9.50
4807:E8	227309	24.79
4807:F8	234764	11.98
4807:G8	246415	11.57
4807:H8	269905	6.20
4807:A9	272275	13.22

4807:B9	276369	14.88
4807:C9	278741	8.68
4807:D9	279834	12.81
4807:E9	284701	17.36
4807:F9	287065	1.65
4807:G9	287495	9.92
4807:H9	288686	4.96
4807:A10	295701	14.46
4807:B10	303244	11.98
4807:C10	303603	-9.92
4807:D10	303800	2.89
4807:E10	304902	10.33
4807:F10	311723	11.16
4807:G10	321484	3.72
4807:H10	331198	4.13
4807:A11	331208	12.40
4807:B11	335649	5.37
4807:C11	338205	7.02
4807:D11	339578	28.93
4807:E11	341902	5.37
4807:F11	342460	9.34
4807:G11	344494	14.05
4807:H11	351110	3.31
4809:A2	31208	6.79
4809:B2	31664	-5.96
4809:C2	31741	0.46
4809:D2	32838	1.83
4809:E2	33005	-9.63
4809:F2	34488	-9.63
4809:G2	34769	-9.17
4809:H2	34774	7.43
4809:A3	34777	-1.83
4809:B3	35676	-7.80
4809:C3	35964	4.59
4809:D3	36425	-3.67
4809:E3	36520	-5.96
4809:F3	36582	-1.93
4809:G3	37003	-3.67
4809:H3	37812	-3.30
4809:A4	38042	4.59
4809:B4	38490	-1.93
4809:C4	38743	5.50
4809:D4	38845	-0.46
4809:E4	38983	-4.13
4809:F4	39336	-3.67
4809:G4	40383	-3.67
4809:H4	40467	10.94
4809:A5	40500	-1.83

4809:B5	40614	-0.92
4809:C5	40669	12.39
4809:D5	41092	-5.96
4809:E5	42014	-4.13
4809:F5	42212	-2.75
4809:G5	44688	-5.96
4809:H5	45153	-11.93
4809:A6	45291	-0.92
4809:B6	46615	-5.05
4809:C6	47617	5.50
4809:D6	47619	7.34
4809:E6	48964	1.38
4809:F6	49652	11.93
4809:G6	49701	-2.75
4809:H6	50405	-11.01
4809:A7	50633	0.00
4809:B7	51331	-1.83
4809:C7	51936	8.26
4809:D7	55770	4.13
4809:E7	57103	-8.62
4809:F7	57165	-1.38
4809:G7	57318	-3.21
4809:H7	57345	-8.26
4809:A8	57794	4.59
4809:B8	58907	-0.46
4809:C8	59776	8.26
4809:D8	60034	6.42
4809:E8	60266	-0.46
4809:F8	60419	2.29
4809:G8	61888	-2.75
4809:H8	61910	11.01
4809:A9	62611	2.29
4809:B9	62665	0.46
4809:C9	63001	4.04
4809:D9	63865	0.92
4809:E9	63963	-9.17
4809:F9	66837	-3.67
4809:G9	67546	-3.67
4809:H9	69421	-5.96
4809:A10	70534	6.88
4809:B10	73170	-2.29
4809:C10	75241	12.39
4809:D10	75885	2.75
4809:E10	77596	-0.92
4809:F10	78130	4.04
4809:G10	79139	-7.34
4809:H10	79253	-1.93
4809:A11	79538	1.38

4809:B11	80141	-5.05
4809:C11	81018	7.34
4809:D11	81120	11.93
4809:E11	81213	-0.46
4809:F11	81660	0.92
4809:G11	81703	-4.59
4809:H11	83715	-10.55
4810:A2	85179	1.53
4810:B2	85326	2.84
4810:C2	87352	3.41
4810:D2	87822	-0.66
4810:E2	88811	0.22
4810:F2	88883	1.97
4810:G2	88962	-4.60
4810:H2	88998	-5.03
4810:A3	89249	2.84
4810:B3	89258	-1.09
4810:C3	89723	0.66
4810:D3	91516	-0.66
4810:E3	92207	8.23
4810:F3	92709	-5.47
4810:G3	92794	10.42
4810:H3	93817	0.66
4810:A4	96491	3.72
4810:B4	98683	-1.53
4810:C4	100120	7.66
4810:D4	101266	-0.22
4810:E4	101777	-3.28
4810:F4	102086	1.09
4810:G4	103770	-1.53
4810:H4	103775	-1.09
4810:A5	106208	14.42
4810:B5	106282	1.97
4810:C5	106461	7.22
4810:D5	106506	0.66
4810:E5	106570	-0.66
4810:F5	106863	-11.16
4810:G5	108235	8.12
4810:H5	108750	-0.66
4810:A6	108753	-9.85
4810:B6	108972	5.91
4810:C6	109084	0.22
4810:D6	109086	5.47
4810:E6	109466	0.22
4810:F6	111552	29.54
4810:G6	112677	1.09
4810:H6	114490	1.09
4810:A7	114831	5.47

4810:B7	117741	1.09
4810:C7	117922	-0.22
4810:D7	118723	12.47
4810:E7	120286	9.41
4810:F7	120307	2.41
4810:G7	120844	-3.72
4810:H7	121781	-5.03
4810:A8	122280	5.91
4810:B8	122297	8.10
4810:C8	122376	6.78
4810:D8	123141	8.97
4810:E8	124146	3.28
4810:F8	125043	8.10
4810:G8	125727	2.84
4810:H8	126405	1.97
4810:A9	126757	-2.47
4810:B9	128141	8.10
4810:C9	128737	7.66
4810:D9	128751	7.22
4810:E9	129260	25.16
4810:F9	57670	13.35
4810:G9	98857	4.60
4810:H9	99796	6.78
4810:A10	102288	10.28
4810:B10	56906	7.66
4810:C10	68982	12.47
4810:D10	71795	12.47
4810:E10	113486	16.85
4810:F10	42846	4.60
4810:G10	50572	2.84
4810:H10	53710	4.60
4810:A11	56455	9.41
4810:B11	70959	5.03
4810:C11	73054	-1.09
4810:D11	101653	16.41
4810:E11	109174	2.84
4810:F11	109719	10.72
4810:G11	117554	7.22
4810:H11	122987	0.66
4811:A2	130872	-2.48
4811:B2	131388	-8.57
4811:C2	131986	9.24
4811:D2	133195	4.76
4811:E2	133356	-2.10
4811:F2	139257	4.38
4811:G2	140892	0.19
4811:H2	143348	-4.76
4811:A3	144958	2.86

4811:B3	144982	-0.86
4811:C3	149046	-0.48
4811:D3	149286	19.24
4811:E3	150954	-5.52
4811:F3	152393	-6.67
4811:G3	152551	-6.67
4811:H3	153365	-7.43
4811:A4	153399	-5.90
4811:B4	154295	2.00
4811:C4	156616	-6.67
4811:D4	157940	8.19
4811:E4	159031	-2.86
4811:F4	163144	-8.95
4811:G4	164208	-7.81
4811:H4	164511	28.00
4811:A5	164678	0.19
4811:B5	165883	-4.00
4811:C5	170578	-1.71
4811:D5	170621	11.62
4811:E5	174027	-10.24
4811:F5	175412	2.10
4811:G5	175415	-0.95
4811:H5	176765	7.81
4811:A6	177989	-1.71
4811:B6	182400	-7.05
4811:C6	190336	-0.95
4811:D6	193043	10.86
4811:E6	196148	7.81
4811:F6	197049	4.00
4811:G6	202883	7.43
4811:H6	204920	6.29
4811:A7	204976	-5.52
4811:B7	205843	-6.67
4811:C7	210816	3.62
4811:D7	215276	17.33
4811:E7	220030	3.62
4811:F7	234945	3.62
4811:G7	236246	-2.67
4811:H7	240502	14.67
4811:A8	255025	-9.71
4811:B8	261037	10.00
4811:C8	261610	-8.95
4811:D8	274905	16.57
4811:E8	277806	10.86
4811:F8	279895	-0.86
4811:G8	282187	11.24
4811:H8	284234	-4.76
4811:A9	288519	-8.19

4811:B9	289365	-2.38
4811:C9	290307	-2.48
4811:D9	292826	15.05
4811:E9	293334	-7.81
4811:F9	294623	7.43
4811:G9	295404	-0.86
4811:H9	298197	-12.95
4811:A10	298793	-10.86
4811:B10	301168	-3.52
4811:C10	309971	-0.19
4811:D10	311074	8.95
4811:E10	311727	14.29
4811:F10	319034	2.48
4811:G10	321506	-3.24
4811:H10	327693	-0.57
4811:A11	329676	-2.86
4811:B11	330497	-5.90
4811:C11	332473	-0.57
4811:D11	335048	-6.67
4811:E11	338106	-3.52
4811:F11	341074	-3.62
4811:G11	343230	100.76
4811:H11	343343	99.14
4812:A2	343344	12.53
4812:B2	343783	13.85
4812:C2	353451	16.48
4812:D2	366807	12.97
4812:E2	367428	16.04
4812:F2	367487	19.56
4812:G2	370367	14.73
4812:H2	372134	10.33
4812:A3	372221	18.24
4812:B3	373427	4.18
4812:C3	373535	16.04
4812:D3	375997	16.48
4812:E3	375392	20.44
4812:F3	379639	49.01
4812:G3	382059	9.45
4812:H3	357683	7.69
4812:A4	403374	8.57
4812:B4	503425	21.32
4812:C4	509563	12.53
4812:D4	515893	14.29
4812:E4	601351	11.65
4812:F4	605333	26.59
4812:G4	622175	3.74
4812:H4	636717	11.21
4812:A5	637290	17.36

4812:B5	638080	10.11
4812:C5	638134	12.53
4812:D5	643150	-9.01
4812:E5	645987	64.84
4812:F5	646976	10.77
4812:G5	659107	5.93
4812:H5	660300	-5.60
4812:A6	479	9.01
4812:B6	1847	9.89
4812:C6	3076	16.92
4812:D6	3193	21.76
4812:E6	3247	19.12
4812:F6	4429	18.24
4812:G6	5426	45.05
4812:H6	5564	-2.42
4812:A7	6137	12.53
4812:B7	6731	16.48
4812:C7	6821	13.85
4812:D7	6844	7.69
4812:E7	7420	16.92
4812:F7	7572	2.86
4812:G7	7745	10.33
4812:H7	7962	2.86
4812:A8	9782	11.65
4812:B8	10173	13.85
4812:C8	10211	15.16
4812:D8	10768	11.65
4812:E8	11296	11.65
4812:F8	227383	15.16
4812:G8	293780	5.49
4812:H8	294154	-6.81
4812:A9	11275	3.30
4812:B9	159632	16.04
4812:C9	170001	16.92
4812:D9	179818	11.65
4812:E9	289090	13.85
4812:F9	294161	18.68
4812:G9	306752	15.60
4812:H9	329284	-1.54
4812:A10	402843	10.33
4812:B10	403379	13.41
4812:C10	647136	3.74
4812:D10	6145	16.04
4812:E10	7218	13.41
4812:F10	7867	14.29
4812:G10	8816	0.22
4812:H10	10865	-3.74
4812:A11	11023	40.22

4812:B11	11276	19.56
4812:C11	148170	73.63
4812:D11	153330	17.80
4812:E11	154587	27.03
4812:F11	157767	8.13
4812:G11	166547	24.40
4812:H11	166583	38.02
4813:A2	11664	7.84
4813:B2	11912	1.92
4813:C2	12028	0.87
4813:D2	12488	1.57
4813:E2	12633	1.22
4813:F2	12644	4.70
4813:G2	12646	7.84
4813:H2	13151	-9.23
4813:A3	13345	8.89
4813:B3	13434	7.84
4813:C3	13579	-0.52
4813:D3	13658	4.01
4813:E3	13800	2.96
4813:F3	14396	2.96
4813:G3	15358	-7.84
4813:H3	15359	3.31
4813:A4	17148	7.14
4813:B4	17362	9.58
4813:C4	19125	1.57
4813:D4	19136	2.96
4813:E4	19141	0.52
4813:F4	19824	1.22
4813:G4	19962	12.72
4813:H4	21333	0.17
4813:A5	21603	3.31
4813:B5	21683	-5.61
4813:C5	21710	-0.52
4813:D5	22801	4.36
4813:E5	22806	1.57
4813:F5	25678	1.92
4813:G5	25740	7.14
4813:H5	28377	0.17
4813:A6	29073	5.40
4813:B6	30930	7.84
4813:C6	31069	1.57
4813:D6	31698	4.36
4813:E6	31703	1.22
4813:F6	34875	1.92
4813:G6	34879	5.40
4813:H6	34910	-7.49
4813:A7	36586	6.10

4813:B7	36753	7.84
4813:C7	36815	1.22
4813:D7	37612	6.45
4813:E7	37955	0.87
4813:F7	38352	3.66
4813:G7	39938	10.03
4813:H7	40275	1.22
4813:A8	40306	3.66
4813:B8	41066	8.19
4813:C8	41376	1.22
4813:D8	41378	5.05
4813:E8	41649	2.96
4813:F8	41805	2.61
4813:G8	42096	19.69
4813:H8	42135	2.96
4813:A9	43308	8.54
4813:B9	43344	9.23
4813:C9	44556	-2.26
4813:D9	45086	5.05
4813:E9	45536	-6.79
4813:F9	45745	0.52
4813:G9	46212	1.22
4813:H9	47522	-2.96
4813:A10	49643	6.45
4813:B10	49847	6.10
4813:C10	50199	-1.92
4813:D10	50680	-1.92
4813:E10	51351	-7.49
4813:F10	52241	-1.57
4813:G10	54860	-1.22
4813:H10	55172	-1.57
4813:A11	55453	5.40
4813:B11	55845	5.75
4813:C11	55862	-5.05
4813:D11	58347	6.52
4813:E11	58724	-1.57
4813:F11	59430	2.61
4813:G11	59782	6.45
4813:H11	59984	-7.14
4814:A2	60303	13.15
4814:B2	61929	3.52
4814:C2	62901	6.11
4814:D2	63161	14.26
4814:E2	66020	17.59
4814:F2	68841	31.67
4814:G2	68971	6.48
4814:H2	70307	-2.78
4814:A3	72947	17.96

4814:B3	73295	8.70
4814:C3	73753	7.96
4814:D3	76549	14.26
4814:E3	76747	22.78
4814:F3	78623	25.74
4814:G3	78697	1.67
4814:H3	82269	0.19
4814:A4	87008	12.78
4814:B4	87690	6.11
4814:C4	88324	5.00
4814:D4	88349	13.15
4814:E4	88402	17.22
4814:F4	88795	34.63
4814:G4	89349	-5.74
4814:H4	89429	-3.15
4814:A5	89759	11.67
4814:B5	92849	6.11
4814:C5	93427	-4.63
4814:D5	93945	17.22
4814:E5	95909	17.59
4814:F5	97865	28.70
4814:G5	98026	2.41
4814:H5	98049	-3.15
4814:A6	54645	12.78
4814:B6	43409	3.89
4814:C6	40749	-10.67
4814:D6	39047	12.41
4814:E6	38007	10.93
4814:F6	98938	7.59
4814:G6	30813	3.52
4814:H6	100058	-3.52
4814:A7	22881	12.04
4814:B7	100942	4.63
4814:C7	21709	0.93
4814:D7	14398	7.96
4814:E7	101298	21.30
4814:F7	13248	23.89
4814:G7	101345	2.78
4814:H7	101679	-2.04
4814:A8	79887	12.04
4814:B8	101758	2.41
4814:C8	33010	-6.48
4814:D8	15784	-2.04
4814:E8	26692	13.15
4814:F8	102554	20.93
4814:G8	103189	-1.30
4814:H8	105432	-5.74
4814:A9	107022	2.78

4814:B9	108783	13.52
4814:C9	109747	-2.78
4814:D9	60037	-2.19
4814:E9	66122	13.52
4814:F9	17055	11.30
4814:G9	92937	-4.26
4814:H9	32892	0.19
4814:A10	20619	9.81
4814:B10	14380	-0.19
4814:C10	33173	-3.52
4814:D10	33182	12.41
4814:E10	11643	14.26
4814:F10	23715	29.07
4814:G10	51349	0.19
4814:H10	17507	2.41
4814:A11	13785	11.67
4814:B11	110899	2.04
4814:C11	14311	1.67
4814:D11	45815	9.07
4814:E11	76478	16.11
4814:F11	20618	29.81
4814:G11	11624	6.85
4814:H11	53934	10.93
4815:A2	112541	-3.51
4815:B2	112547	6.87
4815:C2	112965	-0.46
4815:D2	116508	9.62
4815:E2	116640	-6.56
4815:F2	116644	-2.37
4815:G2	117197	-6.26
4815:H2	117446	1.07
4815:A3	117908	-1.84
4815:B3	118628	8.09
4815:C3	119805	-1.84
4815:D3	120631	7.48
4815:E3	120913	3.82
4815:F3	120961	11.45
4815:G3	121182	5.04
4815:H3	121268	0.46
4815:A4	122253	4.43
4815:B4	126224	3.82
4815:C4	127886	4.73
4815:D4	129220	1.98
4815:E4	129929	-4.12
4815:F4	130801	9.01
4815:G4	130847	-0.46
4815:H4	133002	-1.07
4815:A5	133351	-1.50

4815:B5	134058	16.34
4815:C5	134199	-5.23
4815:D5	135894	-2.90
4815:E5	137399	0.76
4815:F5	137577	-3.51
4815:G5	138389	-2.60
4815:H5	138398	-4.73
4815:A6	139021	-5.04
4815:B6	140873	-0.15
4815:C6	141538	1.37
4815:D6	142269	3.82
4815:E6	144694	9.01
4815:F6	147866	7.48
4815:G6	148832	-2.90
4815:H6	151262	-2.29
4815:A7	153172	7.86
4815:B7	153792	-0.46
4815:C7	154127	5.04
4815:D7	156957	2.90
4815:E7	157522	9.62
4815:F7	158549	-0.76
4815:G7	159686	-7.79
4815:H7	162188	4.73
4815:A8	163802	-6.26
4815:B8	164464	-1.68
4815:C8	165599	5.04
4815:D8	165701	1.98
4815:E8	166596	5.34
4815:F8	166634	4.12
4815:G8	166846	1.37
4815:H8	168221	-6.87
4815:A9	168225	-8.70
4815:B9	170637	3.66
4815:C9	170955	-0.15
4815:D9	174084	1.68
4815:E9	175743	1.37
4815:F9	176367	4.73
4815:G9	177866	-10.37
4815:H9	178873	-7.48
4815:A10	179822	9.92
4815:B10	180964	0.76
4815:C10	193528	-4.12
4815:D10	195327	-4.73
4815:E10	197046	5.34
4815:F10	201634	9.92
4815:G10	203837	2.29
4815:H10	204939	-6.87
4815:A11	205909	-9.92

4815:B11	205913	-10.06
4815:C11	207895	10.15
4815:D11	211336	-8.09
4815:E11	213708	-9.31
4815:F11	214029	-0.15
4815:G11	215275	-7.25
4815:H11	215585	-4.43
4816:A2	216183	57.02
4816:B2	216618	25.07
4816:C2	236254	30.58
4816:D2	228155	66.39
4816:E2	238929	37.19
4816:F2	240029	46.01
4816:G2	241621	54.82
4816:H2	241998	11.29
4816:A3	242557	-6.89
4816:B3	265372	10.74
4816:C3	283856	10.19
4816:D3	285669	-5.79
4816:E3	288024	8.54
4816:F3	289748	3.03
4816:G3	294150	6.72
4816:H3	294625	5.23
4816:A4	294747	-6.89
4816:B4	296934	10.19
4816:C4	299514	6.89
4816:D4	299968	-2.48
4816:E4	301167	9.64
4816:F4	302584	1.93
4816:G4	302867	-3.58
4816:H4	303304	-10.19
4816:A5	305329	6.17
4816:B5	305743	4.13
4816:C5	307703	3.58
4816:D5	308814	27.82
4816:E5	312606	9.53
4816:F5	319029	2.48
4816:G5	319079	0.28
4816:H5	319436	9.09
4816:A6	319471	5.79
4816:B6	321792	10.74
4816:C6	326644	12.95
4816:D6	326921	11.29
4816:E6	327444	49.86
4816:F6	328130	6.89
4816:G6	331972	3.03
4816:H6	332452	-4.68
4816:A7	338564	-7.44

4816:B7	343526	-9.08
4816:C7	343557	10.74
4816:D7	348970	9.09
4816:E7	351674	4.13
4816:F7	351691	9.09
4816:G7	352888	-1.38
4816:H7	358311	-4.68
4816:A8	361570	7.99
4816:B8	364889	9.09
4816:C8	366086	0.83
4816:D8	366802	18.46
4816:E8	367474	17.36
4816:F8	367480	23.42
4816:G8	111847	2.48
4816:H8	125344	-8.54
4816:A9	125605	79.61
4816:B9	135412	98.35
4816:C9	310113	28.93
4816:D9	316458	11.85
4816:E9	318799	18.46
4816:F9	362093	14.05
4816:G9	367416	24.52
4816:H9	140899	-3.13
4816:A10	217306	-0.83
4816:B10	276736	3.03
4816:C10	283845	3.58
4816:D10	294153	3.03
4816:E10	363801	3.03
4816:F10	122385	1.38
4816:G10	134674	-6.34
4816:H10	252172	-1.93
4816:A11	311165	9.64
4816:B11	330796	17.91
4816:C11	149312	-5.79
4816:D11	164880	26.17
4816:E11	269904	6.89
4816:F11	280492	11.29
4816:G11	299967	1.93
4816:H11	331977	4.60
4817:A2	369070	57.19
4817:B2	370383	36.84
4817:C2	372769	46.67
4817:D2	374703	32.28
4817:E2	374814	31.93
4817:F2	375105	36.49
4817:G2	378711	29.12
4817:H2	379468	43.86
4817:A3	379536	40.70

4817:B3	379651	35.09
4817:C3	380279	27.02
4817:D3	400770	29.82
4817:E3	400938	6.67
4817:F3	403268	25.96
4817:G3	408860	99.65
4817:H3	522131	41.05
4817:A4	524615	32.28
4817:B4	525721	32.28
4817:C4	636718	35.09
4817:D4	637343	53.68
4817:E4	637359	31.23
4817:F4	637827	42.46
4817:G4	638636	34.04
4817:H4	641396	37.19
4817:A5	643029	35.44
4817:B5	645330	50.53
4817:C5	661221	40.70
4817:D5	3753	22.11
4817:E5	5053	22.46
4817:F5	5476	28.07
4817:G5	6101	24.21
4817:H5	6268	28.42
4817:A6	7436	20.70
4817:B6	7578	30.53
4817:C6	9037	62.81
4817:D6	12544	30.88
4817:E6	12628	25.96
4817:F6	12650	2.46
4817:G6	13156	26.67
4817:H6	13176	24.91
4817:A7	14506	22.11
4817:B7	16722	-7.02
4817:C7	17128	30.53
4817:D7	18883	41.40
4817:E7	24032	65.96
4817:F7	24951	24.56
4817:G7	25435	23.51
4817:H7	25457	29.12
4817:A8	26349	21.40
4817:B8	26980	20.00
4817:C8	27305	37.89
4817:D8	28080	26.32
4817:E8	30260	19.65
4817:F8	32673	74.04
4817:G8	32873	21.05
4817:H8	33353	41.05
4817:A9	33478	30.53

4817:B9	33738	15.79
4817:C9	34219	24.21
4817:D9	34865	20.35
4817:E9	35545	22.46
4817:F9	35582	19.30
4817:G9	36525	20.70
4817:H9	36693	16.14
4817:A10	36758	79.30
4817:B10	36923	17.19
4817:C10	37168	41.75
4817:D10	37187	46.32
4817:E10	38090	24.91
4817:F10	39984	22.46
4817:G10	40269	20.00
4817:H10	41098	81.05
4817:A11	43088	21.40
4817:B11	43271	24.56
4817:C11	43506	6.67
4817:D11	43998	18.95
4817:E11	44584	28.07
4817:F11	44750	97.89
4817:G11	45527	4.21
4817:H11	46213	60.35
4818:A2	46492	37.34
4818:B2	47680	12.94
4818:C2	48388	13.31
4818:D2	48443	87.99
4818:E2	50648	9.98
4818:F2	50651	24.40
4818:G2	50690	57.30
4818:H2	51683	14.42
4818:A3	53874	10.72
4818:B3	55152	14.05
4818:C3	56410	10.35
4818:D3	60013	25.51
4818:E3	60183	2.22
4818:F3	60423	4.07
4818:G3	61642	6.62
4818:H3	62375	7.39
4818:A4	63543	-3.33
4818:B4	64672	4.07
4818:C4	65689	8.13
4818:D4	68116	9.98
4818:E4	69359	15.53
4818:F4	70413	6.28
4818:G4	70895	4.07
4818:H4	71097	13.31
4818:A5	71866	-1.48

4818:B5	71881	10.35
4818:C5	73254	6.65
4818:D5	76015	10.72
4818:E5	76988	10.35
4818:F5	79486	2.59
4818:G5	79559	8.85
4818:H5	81750	15.16
4818:A6	82560	9.54
4818:B6	83497	17.01
4818:C6	86467	7.02
4818:D6	87010	14.05
4818:E6	87084	7.76
4818:F6	87136	11.09
4818:G6	87838	9.61
4818:H6	90749	12.20
4818:A7	91378	3.70
4818:B7	91382	2.96
4818:C7	92892	7.39
4818:D7	93033	16.27
4818:E7	95204	7.02
4818:F7	95916	4.44
4818:G7	524385	1.85
4818:H7	680515	5.90
4818:A8	689002	0.74
4818:B8	1014	43.99
4818:C8	11437	-4.44
4818:D8	12262	18.48
4818:E8	13616	1.11
4818:F8	14142	6.28
4818:G8	22070	18.85
4818:H8	30205	-8.87
4818:A9	33570	8.13
4818:B9	94600	9.98
4818:C9	408734	3.64
4818:D9	369066	72.83
4818:E9	11307	5.91
4818:F9	25673	6.65
4818:G9	26112	5.91
4818:H9	26113	-4.07
4818:A10	30622	8.50
4818:B10	33575	6.65
4818:C10	66695	8.13
4818:D10	81493	7.02
4818:E10	636734	7.39
4818:F10	680516	-4.44
4818:G10	3064	61.37
4818:H10	19123	3.70
4818:A11	45545	-1.85

4818:B11	80313	22.55
4818:C11	653004	8.13
4818:D11	48617	11.09
4818:E11	60659	4.81
4818:F11	64859	8.13
4818:G11	81856	62.48
4818:H11	83961	4.81
4819:A2	96541	17.20
4819:B2	96996	5.02
4819:C2	99634	2.87
4819:D2	99660	8.24
4819:E2	99663	2.87
4819:F2	101789	19.00
4819:G2	105348	19.71
4819:H2	105781	-2.87
4819:A3	105798	6.81
4819:B3	105827	23.30
4819:C3	106464	5.38
4819:D3	110332	7.53
4819:E3	110562	2.51
4819:F3	111194	-1.43
4819:G3	112125	4.30
4819:H3	112203	5.73
4819:A4	114997	-0.72
4819:B4	116397	4.66
4819:C4	120290	6.09
4819:D4	120622	4.66
4819:E4	121908	4.66
4819:F4	123389	16.49
4819:G4	126226	6.45
4819:H4	126347	7.17
4819:A5	129536	-0.72
4819:B5	135184	8.24
4819:C5	137112	4.30
4819:D5	142277	4.30
4819:E5	143974	3.94
4819:F5	146071	9.68
4819:G5	146554	5.38
4819:H5	146770	4.66
4819:A6	147358	-1.08
4819:B6	149054	7.53
4819:C6	153391	6.45
4819:D6	154585	5.38
4819:E6	156563	2.19
4819:F6	157725	2.15
4819:G6	158959	12.90
4819:H6	159092	6.09
4819:A7	159398	3.94

4819:B7	164435	27.24
4819:C7	164459	3.58
4819:D7	165704	7.53
4819:E7	169409	5.38
4819:F7	176736	22.94
4819:G7	177407	6.09
4819:H7	186067	3.23
4819:A8	186194	-5.02
4819:B8	186200	7.17
4819:C8	190501	25.45
4819:D8	191441	6.45
4819:E8	194308	6.81
4819:F8	201659	3.58
4819:G8	201989	-5.38
4819:H8	202705	16.49
4819:A9	205827	0.00
4819:B9	205842	8.60
4819:C9	205912	5.38
4819:D9	211340	7.53
4819:E9	211787	-2.15
4819:F9	215684	7.96
4819:G9	215689	-3.66
4819:H9	216607	9.32
4819:A10	216621	2.87
4819:B10	216623	2.87
4819:C10	228137	13.98
4819:D10	228150	83.87
4819:E10	241619	6.09
4819:F10	241624	2.87
4819:G10	244387	7.53
4819:H10	246999	-0.72
4819:A11	270063	6.45
4819:B11	270916	6.81
4819:C11	281307	-2.15
4819:D11	281383	1.43
4819:E11	281623	-4.66
4819:F11	281624	-6.09
4819:G11	282137	5.73
4819:H11	283849	2.51
4820:A2	288387	75.76
4820:B2	293360	32.40
4820:C2	294750	4.43
4820:D2	294756	14.69
4820:E2	295300	10.96
4820:F2	299119	12.82
4820:G2	300540	53.38
4820:H2	303294	9.56
4820:A3	303612	10.02

4820:B3	305780	37.06
4820:C3	308848	-1.89
4820:D3	308849	-7.69
4820:E3	319012	7.69
4820:F3	319424	8.16
4820:G3	319449	7.23
4820:H3	321517	16.55
4820:A4	326182	60.84
4820:B4	326184	45.45
4820:C4	326385	12.82
4820:D4	326422	28.67
4820:E4	326757	10.96
4820:F4	328010	9.56
4820:G4	328111	25.41
4820:H4	329052	8.62
4820:A5	329249	54.78
4820:B5	329255	60.84
4820:C5	330770	23.54
4820:D5	332670	68.30
4820:E5	333544	9.56
4820:F5	335504	8.16
4820:G5	337726	5.83
4820:H5	339589	5.83
4820:A6	339594	6.29
4820:B6	339630	6.29
4820:C6	341956	14.69
4820:D6	343550	15.15
4820:E6	345850	8.16
4820:F6	346578	-3.50
4820:G6	349156	0.70
4820:H6	352890	3.03
4820:A7	362639	2.56
4820:B7	366289	26.81
4820:C7	369986	9.56
4820:D7	371765	18.41
4820:E7	372146	17.95
4820:F7	372275	36.13
4820:G7	372287	6.29
4820:H7	98363	84.62
4820:A8	114449	7.32
4820:B8	146769	6.29
4820:C8	187675	11.42
4820:D8	201868	15.15
4820:E8	205832	17.95
4820:F8	217913	29.60
4820:G8	343549	16.55
4820:H8	366801	17.48
4820:A9	252359	8.62

4820:B9	321502	31.93
4820:C9	324623	1.63
4820:D9	135381	8.16
4820:E9	142446	49.65
4820:F9	149050	3.03
4820:G9	211356	3.96
4820:H9	331968	17.95
4820:A10	99657	23.08
4820:B10	111118	7.69
4820:C10	133075	6.29
4820:D10	150114	49.18
4820:E10	222362	8.16
4820:F10	278323	17.02
4820:G10	326375	16.55
4820:H10	329250	69.23
4820:A11	338578	3.50
4820:B11	339316	10.02
4820:C11	135168	10.96
4820:D11	145992	8.16
4820:E11	197008	7.23
4820:F11	209901	19.81
4820:G11	245091	7.23
4820:H11	300289	13.29
4821:A2	372499	17.79
4821:B2	375981	13.52
4821:C2	375982	21.35
4821:D2	378717	17.79
4821:E2	379538	26.69
4821:F2	379697	25.27
4821:G2	401077	12.81
4821:H2	403447	18.51
4821:A3	407628	17.44
4821:B3	622608	37.37
4821:C3	623109	11.74
4821:D3	623638	30.25
4821:E3	630602	53.02
4821:F3	631160	22.42
4821:G3	632536	12.81
4821:H3	634396	55.16
4821:A4	637153	34.52
4821:B4	637317	13.52
4821:C4	637325	17.44
4821:D4	651084	23.84
4821:E4	660151	8.54
4821:F4	3323	18.15
4821:G4	5157	9.96
4821:H4	5836	24.91
4821:A5	7419	10.68

4821:B5	9032	36.30
4821:C5	11668	8.54
4821:D5	13487	25.98
4821:E5	14974	16.01
4821:F5	16437	9.61
4821:G5	16736	4.09
4821:H5	17355	22.42
4821:A6	19061	17.08
4821:B6	20192	-4.98
4821:C6	24113	22.06
4821:D6	29200	24.56
4821:E6	31748	15.66
4821:F6	34871	10.32
4821:G6	36317	10.68
4821:H6	36818	15.66
4821:A7	46075	19.22
4821:B7	46385	13.88
4821:C7	49852	-3.91
4821:D7	50650	67.97
4821:E7	50654	71.89
4821:F7	50688	62.63
4821:G7	56287	35.23
4821:H7	57624	37.37
4821:A8	63680	18.86
4821:B8	65537	11.03
4821:C8	70799	17.44
4821:D8	76350	14.95
4821:E8	78846	12.46
4821:F8	80137	10.32
4821:G8	81463	12.46
4821:H8	81915	19.93
4821:A9	84100	75.09
4821:B9	89602	43.42
4821:C9	91340	21.35
4821:D9	91355	22.06
4821:E9	91356	20.28
4821:F9	91357	21.35
4821:G9	91368	17.08
4821:H9	91396	23.49
4821:A10	91397	65.84
4821:B10	97920	44.48
4821:C10	99925	21.00
4821:D10	100708	18.51
4821:E10	102314	15.66
4821:F10	103331	51.96
4821:G10	106231	17.08
4821:H10	107679	27.76
4821:A11	110300	23.49

4821:B11	111210	17.08
4821:C11	114414	37.01
4821:D11	116709	24.91
4821:E11	117028	62.99
4821:F11	117268	18.15
4821:G11	117987	11.74
4821:H11	118818	11.74
4822:A2	120289	4.73
4822:B2	124818	27.70
4822:C2	126837	0.34
4822:D2	131467	3.72
4822:E2	131616	-4.73
4822:F2	134137	-1.82
4822:G2	139168	-5.07
4822:H2	151721	5.07
4822:A3	151888	5.54
4822:B3	154389	3.38
4822:C3	156565	-8.45
4822:D3	159566	20.95
4822:E3	163639	27.03
4822:F3	163823	1.01
4822:G3	164991	16.55
4822:H3	166637	1.69
4822:A4	191454	-6.08
4822:B4	201863	55.41
4822:C4	204262	14.19
4822:D4	204665	2.70
4822:E4	214009	60.14
4822:F4	215721	22.64
4822:G4	216606	4.05
4822:H4	217697	-3.38
4822:A5	250429	-2.70
4822:B5	263220	-4.39
4822:C5	270071	2.03
4822:D5	271923	3.72
4822:E5	275266	-5.41
4822:F5	275971	-8.11
4822:G5	280594	-4.39
4822:H5	289359	-4.73
4822:A6	292140	-4.73
4822:B6	292923	-5.07
4822:C6	293962	-3.04
4822:D6	295486	10.14
4822:E6	298892	17.57
4822:F6	347463	-6.42
4822:G6	309401	-9.12
4822:H6	310354	-5.74
4822:A7	317605	-9.46

4822:B7	319435	-5.07
4822:C7	319994	21.96
4822:D7	320218	55.74
4822:E7	325014	9.12
4822:F7	329065	-8.11
4822:G7	338519	17.70
4822:H7	339161	31.42
4822:A8	11881	11.15
4822:B8	12666	-5.41
4822:C8	37219	-5.41
4822:D8	116702	2.36
4822:E8	177862	11.15
4822:F8	37627	4.05
4822:G8	321491	-3.72
4822:H8	345845	0.00
4822:A9	637578	4.05
4822:B9	32984	-7.43
4822:C9	45572	-8.78
4822:D9	73053	15.34
4822:E9	216633	7.77
4822:F9	372767	1.01
4822:G9	622691	17.23
4822:H9	680495	26.69
4822:A10	4292	30.07
4822:B10	62685	-7.43
4822:C10	84126	10.41
4822:D10	88600	42.91
4822:E10	88916	9.46
4822:F10	125095	28.72
4822:G10	327702	-5.74
4822:H10	13316	2.70
4822:A11	54709	-9.80
4822:B11	60785	20.95
4822:C11	99867	4.73
4822:D11	142335	7.09
4822:E11	163443	3.72
4822:F11	275428	-5.74
4822:G11	319709	8.11
4822:H11	338042	12.84
4823:A2	354261	22.01
4823:B2	359472	48.33
4823:C2	367306	47.37
4823:D2	367469	8.61
4823:E2	379388	-2.39
4823:F2	379555	-9.57
4823:G2	382035	-10.96
4823:H2	601359	-5.26
4823:A3	603071	3.40

4823:B3	607097	6.22
4823:C3	622689	17.70
4823:D3	645033	-10.00
4823:E3	661122	-10.88
4823:F3	670283	12.92
4823:G3	672865	3.16
4823:H3	1614	-6.75
4823:A4	5856	58.85
4823:B4	12865	-8.61
4823:C4	13051	43.06
4823:D4	13294	55.98
4823:E4	13791	-4.78
4823:F4	30625	2.11
4823:G4	41148	-5.26
4823:H4	42199	-2.10
4823:A5	53275	12.92
4823:B5	57608	-2.39
4823:C5	58904	-8.13
4823:D5	59620	-6.70
4823:E5	65238	13.40
4823:F5	70933	-8.61
4823:G5	85433	-1.48
4823:H5	89201	-6.22
4823:A6	89821	6.22
4823:B6	105584	-4.78
4823:C6	107582	10.53
4823:D6	107677	-9.57
4823:E6	123418	-5.79
4823:F6	123527	-1.44
4823:G6	127133	7.70
4823:H6	128606	5.31
4823:A7	136513	3.83
4823:B7	143241	-2.39
4823:C7	156516	55.50
4823:D7	163910	36.84
4823:E7	164676	3.35
4823:F7	172255	0.00
4823:G7	201631	-9.09
4823:H7	204232	69.86
4823:A8	215718	42.11
4823:B8	234348	3.35
4823:C8	280058	-2.87
4823:D8	290311	-1.91
4823:E8	295358	19.62
4823:F8	305798	2.49
4823:G8	309892	42.58
4823:H8	311153	11.00
4823:A9	321496	39.71

4823:B9	328403	0.48
4823:C9	335506	-9.09
4823:D9	337832	2.39
4823:E9	373600	4.31
4823:F9	376254	-3.83
4823:G9	378719	14.83
4823:H9	379099	45.45
4823:A10	8675	68.90
4823:B10	11667	40.19
4823:C10	15910	-2.39
4823:D10	19803	-10.05
4823:E10	36508	-3.35
4823:F10	37553	13.40
4823:G10	37641	-4.78
4823:H10	41400	2.39
4823:A11	55691	64.59
4823:B11	64876	17.22
4823:C11	67436	11.48
4823:D11	70931	7.18
4823:E11	73735	56.46
4823:F11	80997	45.45
4823:G11	103520	47.37
4823:H11	107522	-7.18
4824:A2	116339	8.28
4824:B2	146771	49.56
4824:C2	166375	18.98
4824:D2	168184	20.04
4824:E2	196515	19.33
4824:F2	211490	17.93
4824:G2	281816	14.06
4824:H2	292253	16.87
4824:A3	317003	12.30
4824:B3	319990	68.89
4824:C3	322661	25.31
4824:D3	335979	28.82
4824:E3	342459	29.17
4824:F3	343256	14.76
4824:G3	371178	18.28
4824:H3	379696	19.33
4824:A4	31762	40.07
4824:B4	45384	16.87
4824:C4	61610	62.92
4824:D4	80731	77.68
4824:E4	80735	57.64
4824:F4	91529	15.47
4824:G4	133071	15.47
4824:H4	139105	15.11
4824:A5	202386	61.86

4824:B5	260594	34.09
4824:C5	345647	35.50
4824:D5	354844	-5.98
4824:E5	654260	13.01
4824:F5	679525	67.49
4824:G5	727038	54.83
4824:H5	143491	5.62
4824:A6	177365	47.10
4824:B6	268251	65.38
4824:C6	330500	25.31
4824:D6	19990	2.81
4824:E6	122819	23.90
4824:F6	227186	13.71
4824:G6	11926	4.22
4824:H6	56779	20.74
4824:A7	59814	27.77
4824:B7	133114	28.82
4824:C7	373981	-1.05
4824:D7	614826	26.01
4824:E7	350187	20.74
4824:F7	341196	99.47
4824:G7	168027	18.98
4824:H7	3391	20.74
4824:A8	178249	21.79
4824:B8	30663	27.77
4824:C8	222365	22.85
4824:D8	328087	25.66
4824:E8	665497	21.09
4824:F8	107701	39.02
4824:G8	158413	24.96
4824:H8	309874	55.54
4824:A9	19970	25.66
4824:B9	96021	23.55
4824:C9	380802	75.92
4824:D9	121868	25.66
4824:E9	203912	48.15
4824:F9	638432	35.50
4824:G9	5907	16.17
4824:H9	277184	5.98
4824:A10	639174	21.79
4824:B10	109128	24.60
4824:C10	166259	23.55
4824:D10	60339	30.93
4824:E10	146557	39.37
4824:F10	159242	22.14
4824:G10	308835	23.90
4824:H10	310325	63.27
4824:A11	21970	36.56

Mechanistic Set

Plate ID: Well ID	NSC number	% Inhibition
4790:A2	32065	-4.53
4790:B2	3970	12.25
4790:C2	4728	10.91
4790:D2	4960	13.93
4790:E2	5354	12.92
4790:F2	89303	9.23
4790:G2	295156	11.58
4790:H2	118976	4.53
4790:A3	261726	-3.86
4790:B3	285166	11.24
4790:C3	626433	12.92
4790:D3	635968	17.95
4790:E3	749	13.59
4790:F3	755	15.27
4790:G3	11779	11.24
4790:H3	18804	8.89
4790:A4	18805	-0.17
4790:B4	37364	15.27
4790:C4	349438	12.25
4790:D4	400944	59.56
4790:E4	603719	11.24
4790:F4	614928	11.91
4790:G4	695218	5.54
4790:H4	3852	-1.44
4790:A5	4170	16.61
4790:B5	4857	22.65
4790:C5	11897	9.56
4790:D5	45388	17.28
4790:E5	51148	52.18
4790:F5	54297	17.95
4790:G5	99733	10.57
4790:H5	111041	84.40
4790:A6	326397	13.93
4790:B6	369317	6.54
4790:C6	369318	11.58
4790:D6	625639	19.30
4790:E6	61805	19.97
4790:F6	95580	21.98
4790:G6	95678	-1.85
4790:H6	98447	15.27
4790:A7	148958	14.60
4790:B7	176324	22.65
4790:C7	620050	18.62
4790:D7	625748	66.61
4790:E7	664298	10.57

4790:F7	673912	10.57
4790:G7	1620	6.88
4790:H7	4280	13.26
4790:A8	26045	8.56
4790:B8	107415	10.23
4790:C8	128734	6.21
4790:D8	132493	5.54
4790:E8	166464	9.23
4790:F8	357683	10.57
4790:G8	403883	12.92
4790:H8	606532	-7.01
4790:A9	635441	3.52
4790:B9	635975	9.23
4790:C9	657449	5.20
4790:D9	666526	8.22
4790:E9	688795	14.26
4790:F9	693053	15.27
4790:G9	1771	75.00
4790:H9	3905	8.89
4790:A10	5200	3.72
4790:B10	7210	9.56
4790:C10	7833	32.72
4790:D10	11905	11.91
4790:E10	18938	7.55
4790:F10	21548	17.28
4790:G10	33004	11.91
4790:H10	56544	2.92
4790:A11	66914	8.22
4790:B11	86100	10.23
4790:C11	87206	13.59
4790:D11	99027	3.86
4790:E11	126849	11.24
4790:F11	169543	4.66
4790:G11	175634	9.56
4790:H11	299879	12.92
4791:A2	319726	-1.39
4791:B2	328587	-8.08
4791:C2	373853	-3.06
4791:D2	375294	5.85
4791:E2	375575	16.99
4791:F2	379531	11.98
4791:G2	602617	15.32
4791:H2	607347	15.88
4791:A3	625355	1.95
4791:B3	635563	-1.31
4791:C3	637833	7.52
4791:D3	645987	32.59
4791:E3	664286	14.21

4791:F3	664331	27.02
4791:G3	674495	16.99
4791:H3	697726	19.78
4791:A4	186	0.28
4791:B4	750	18.66
4791:C4	1906	54.32
4791:D4	4114	1.95
4791:E4	10447	26.46
4791:F4	13966	11.42
4791:G4	22194	16.99
4791:H4	29603	8.08
4791:A5	32946	9.19
4791:B5	63984	14.76
4791:C5	80087	20.33
4791:D5	85236	29.25
4791:E5	85998	16.99
4791:F5	106296	20.33
4791:G5	119686	-9.19
4791:H5	145150	15.88
4791:A6	146268	10.31
4791:B6	146604	19.22
4791:C6	175274	17.55
4791:D6	176655	25.35
4791:E6	191384	26.46
4791:F6	191389	31.48
4791:G6	267213	17.55
4791:H6	278619	14.21
4791:A7	283162	7.52
4791:B7	286193	12.53
4791:C7	292147	6.41
4791:D7	307454	22.56
4791:E7	310618	33.70
4791:F7	320846	2.65
4791:G7	336628	15.88
4791:H7	382007	44.85
4791:A8	400978	2.51
4791:B8	405158	15.32
4791:C8	634471	30.92
4791:D8	634650	25.35
4791:E8	643028	15.32
4791:F8	643031	22.56
4791:G8	659501	28.13
4791:H8	663996	20.89
4791:A9	667235	12.53
4791:B9	676561	5.29
4791:C9	7364	23.12
4791:D9	9856	13.65
4791:E9	65381	30.92

4791:F9	40212	43.73
4791:G9	253272	30.36
4791:H9	63446	20.33
4791:A10	118742	13.65
4791:B10	54650	16.43
4791:C10	105808	29.25
4791:D10	637729	6.96
4791:E10	667251	4.74
4791:F10	1026	27.02
4791:G10	529469	18.11
4791:H10	347466	20.89
4791:A11	9706	14.76
4791:B11	159935	30.92
4791:C11	296961	26.46
4791:D11	326231	13.09
4791:E11	174280	11.42
4791:F11	163501	14.21
4791:G11	15200	19.78
4791:H11	263500	28.13
4792:A2	13973	8.87
4792:B2	19994	13.24
4792:C2	22842	13.80
4792:D2	22992	8.73
4792:E2	33006	16.62
4792:F2	35866	11.55
4792:G2	49660	10.42
4792:H2	63878	0.28
4792:A3	69187	-9.30
4792:B3	70422	9.86
4792:C3	71851	14.37
4792:D3	74420	9.86
4792:E3	76747	41.41
4792:F3	80396	14.37
4792:G3	82116	7.04
4792:H3	89671	8.73
4792:A4	97703	7.75
4792:B4	126771	-7.61
4792:C4	129943	13.80
4792:D4	139109	13.24
4792:E4	145669	-2.54
4792:F4	154754	9.86
4792:G4	155595	9.30
4792:H4	169779	3.10
4792:A5	175493	56.62
4792:B5	185065	-0.28
4792:C5	191392	8.73
4792:D5	191393	12.68
4792:E5	208914	14.93

4792:F5	264880	12.11
4792:G5	269142	11.55
4792:H5	284356	14.37
4792:A6	308847	2.82
4792:B6	404241	16.06
4792:C6	406021	7.04
4792:D6	408120	4.37
4792:E6	605756	-7.32
4792:F6	621889	7.61
4792:G6	622627	21.69
4792:H6	622640	6.48
4792:A7	622684	1.97
4792:B7	625641	3.38
4792:C7	627708	4.23
4792:D7	632233	32.96
4792:E7	635404	-5.77
4792:F7	636817	41.97
4792:G7	637731	-3.66
4792:H7	639754	7.04
4792:A8	640624	-8.17
4792:B8	643174	3.94
4792:C8	643186	17.75
4792:D8	643910	16.06
4792:E8	654705	9.86
4792:F8	658144	17.18
4792:G8	687667	-8.73
4792:H8	693632	2.82
4792:A9	697923	36.90
4792:B9	12825	-1.97
4792:C9	31702	36.90
4792:D9	36826	9.86
4792:E9	45575	12.11
4792:F9	63701	7.75
4792:G9	79456	3.80
4792:H9	95848	10.42
4792:A10	105014	-9.86
4792:B10	119875	54.93
4792:C10	168221	7.61
4792:D10	175296	14.37
4792:E10	224117	10.42
4792:F10	233872	10.99
4792:G10	267033	8.17
4792:H10	292684	10.99
4792:A11	349155	-5.35
4792:B11	407010	4.08
4792:C11	407335	7.04
4792:D11	610744	15.49
4792:E11	611750	12.11

4792:F11	622616	8.17
4792:G11	625487	9.86
4792:H11	629301	21.69
4793:A2	630374	-5.57
4793:B2	631583	0.00
4793:C2	634863	12.26
4793:D2	636786	13.93
4793:E2	643175	-2.06
4793:F2	643774	-6.69
4793:G2	650573	-0.56
4793:H2	652287	6.69
4793:A3	658709	7.80
4793:B3	659997	19.50
4793:C3	662553	-3.90
4793:D3	666168	-2.17
4793:E3	25149	13.37
4793:F3	34757	-1.59
4793:G3	38721	7.80
4793:H3	65346	-3.90
4793:A4	68093	78.55
4793:B4	78365	6.13
4793:C4	79451	-2.07
4793:D4	82025	-5.01
4793:E4	92510	11.70
4793:F4	104801	15.04
4793:G4	106995	8.97
4793:H4	128305	4.48
4793:A5	147340	5.01
4793:B5	154020	3.90
4793:C5	168415	14.48
4793:D5	175636	16.16
4793:E5	202000	16.16
4793:F5	208913	18.38
4793:G5	266535	16.71
4793:H5	267461	26.18
4793:A6	328477	-3.37
4793:B6	329279	-5.13
4793:C6	330770	6.13
4793:D6	352890	16.71
4793:E6	616232	15.04
4793:F6	618332	92.48
4793:G6	619165	13.37
4793:H6	620277	12.26
4793:A7	622732	2.61
4793:B7	625483	15.60
4793:C7	625590	16.71
4793:D7	629659	15.60
4793:E7	631152	27.30

4793:F7	631160	23.40
4793:G7	633001	15.04
4793:H7	637914	11.14
4793:A8	664327	-0.56
4793:B8	667467	4.46
4793:C8	680506	15.60
4793:D8	682864	17.27
4793:E8	1027	98.61
4793:F8	4810	1.11
4793:G8	18891	13.93
4793:H8	36693	9.47
4793:A9	47147	19.50
4793:B9	80756	5.57
4793:C9	93739	8.36
4793:D9	106408	17.27
4793:E9	118735	29.53
4793:F9	140911	77.44
4793:G9	224124	58.50
4793:H9	680516	6.80
4793:A10	59269	6.13
4793:B10	204985	7.24
4793:C10	640391	9.47
4793:D10	224131	15.04
4793:E10	65937	12.81
4793:F10	41809	11.14
4793:G10	635140	17.83
4793:H10	157004	6.69
4793:A11	28002	-0.56
4793:B11	634224	15.04
4793:C11	39202	17.27
4793:D11	60309	5.57
4793:E11	636132	5.57
4793:F11	118732	55.15
4793:G11	338259	72.42
4793:H11	651079	94.71
4794:A2	282752	-2.14
4794:B2	294577	10.22
4794:C2	294961	45.37
4794:D2	299187	-7.67
4794:E2	305782	-3.83
4794:F2	321803	12.78
4794:G2	335142	1.92
4794:H2	338720	13.42
4794:A3	339004	0.64
4794:B3	349156	17.25
4794:C3	360861	24.28
4794:D3	376791	15.34
4794:E3	603108	8.31

4794:F3	621094	5.85
4794:G3	622608	8.31
4794:H3	622690	7.89
4794:A4	623135	-7.25
4794:B4	623637	-1.28
4794:C4	624161	8.31
4794:D4	624358	99.60
4794:E4	632536	7.03
4794:F4	632839	3.83
4794:G4	632841	-6.39
4794:H4	634658	1.92
4794:A5	635121	1.28
4794:B5	635306	31.95
4794:C5	635337	20.45
4794:D5	636084	15.34
4794:E5	637578	33.23
4794:F5	643164	-5.75
4794:G5	648422	12.14
4794:H5	651080	5.75
4794:A6	658285	7.32
4794:B6	658388	-3.83
4794:C6	664329	3.83
4794:D6	11926	-3.19
4794:E6	20514	-3.19
4794:F6	44690	8.31
4794:G6	53908	8.95
4794:H6	61811	7.25
4794:A7	123390	6.39
4794:B7	135996	21.09
4794:C7	143648	17.89
4794:D7	157930	2.56
4794:E7	173905	35.78
4794:F7	178249	-0.64
4794:G7	241509	8.95
4794:H7	257473	7.67
4794:A8	313981	99.60
4794:B8	316157	7.03
4794:C8	320864	7.67
4794:D8	323241	9.58
4794:E8	376265	-8.95
4794:F8	382766	33.23
4794:G8	383468	17.89
4794:H8	600305	2.78
4794:A9	616355	-1.92
4794:B9	620358	44.09
4794:C9	631529	84.35
4794:D9	633209	47.28
4794:E9	634232	28.12

4794:F9	634396	53.67
4794:G9	637680	14.70
4794:H9	643162	3.19
4794:A10	643163	5.78
4794:B10	646189	69.65
4794:C10	646200	-0.64
4794:D10	647613	24.92
4794:E10	651084	5.11
4794:F10	678932	7.67
4794:G10	684845	20.45
4794:H10	689872	61.34
4794:A11	1011	42.81
4794:B11	14574	-2.78
4794:C11	24113	25.56
4794:D11	26273	9.58
4794:E11	32982	-8.18
4794:F11	36437	14.06
4794:G11	83265	3.83
4794:H11	97911	-3.19
4795:A2	115538	2.93
4795:B2	123115	-4.23
4795:C2	140377	36.81
4795:D2	157389	-2.28
4795:E2	163088	10.29
4795:F2	174163	90.23
4795:G2	671424	-8.11
4795:H2	185056	4.23
4795:A3	211500	-4.89
4795:B3	241906	0.33
4795:C3	265459	33.55
4795:D3	302979	17.26
4795:E3	314622	-1.63
4795:F3	327697	5.54
4795:G3	343513	4.20
4795:H3	352876	-1.63
4795:A4	407806	-0.33
4795:B4	604535	2.28
4795:C4	614826	-0.33
4795:D4	617540	-8.14
4795:E4	620279	5.31
4795:F4	626734	7.49
4795:G4	629713	2.48
4795:H4	631521	88.27
4795:A5	635437	0.98
4795:B5	635448	74.59
4795:C5	635542	22.48
4795:D5	640580	47.23
4795:E5	645033	-4.89

4795:F5	647363	6.19
4795:G5	681730	10.75
4795:H5	681741	0.98
4795:A6	377	-2.28
4795:B6	757	-2.93
4795:C6	14974	-2.93
4795:D6	24817	2.28
4795:E6	62791	-0.33
4795:F6	77021	-4.23
4795:G6	98542	2.70
4795:H6	104117	3.29
4795:A7	126727	2.93
4795:B7	166381	5.96
4795:C7	169600	48.53
4795:D7	172946	1.63
4795:E7	249992	6.81
4795:F7	262665	3.55
4795:G7	268986	2.28
4795:H7	281245	4.23
4795:A8	302358	6.19
4795:B8	305884	6.84
4795:C8	322069	16.61
4795:D8	403148	3.58
4795:E8	603577	53.75
4795:F8	624169	4.89
4795:G8	634473	0.98
4795:H8	635321	-4.89
4795:A9	635326	-0.98
4795:B9	337612	1.40
4795:C9	642649	2.93
4795:D9	182986	-2.93
4795:E9	601101	1.63
4795:F9	136037	4.23
4795:G9	621486	5.54
4795:H9	629971	-2.28
4795:A10	634568	6.84
4795:B10	293927	12.70
4795:C10	69852	4.89
4795:D10	99016	3.58
4795:E10	668270	26.38
4795:F10	672904	23.13
4795:G10	26040	-0.33
4795:H10	235082	3.55
4795:A11	5890	-5.54
4795:B11	284751	-0.33
4795:C11	329277	-3.58
4795:D11	146397	-9.45
4795:E11	163443	-10.75

4795:F11	371846	4.89
4795:G11	623051	2.28
4795:H11	623059	-2.93
4796:A2	635328	5.88
4796:B2	635435	7.42
4796:C2	635438	2.30
4796:D2	644735	-12.53
4796:E2	657456	12.02
4796:F2	678917	12.53
4796:G2	680509	6.39
4796:H2	684480	20.72
4796:A3	705330	7.93
4796:B3	740	2.81
4796:C3	2186	33.50
4796:D3	2979	13.04
4796:E3	11930	1.28
4796:F3	19857	1.79
4796:G3	24048	12.53
4796:H3	24818	7.93
4796:A4	24819	16.11
4796:B4	33410	9.97
4796:C4	35489	10.49
4796:D4	35949	23.27
4796:E4	40341	39.13
4796:F4	40666	2.81
4796:G4	43321	5.37
4796:H4	47438	6.39
4796:A5	51812	-7.93
4796:B5	66300	11.00
4796:C5	67580	6.91
4796:D5	67690	8.44
4796:E5	71300	6.39
4796:F5	71669	2.30
4796:G5	73413	-4.86
4796:H5	85561	42.71
4796:A6	85700	-5.37
4796:B6	98904	7.42
4796:C6	100856	11.00
4796:D6	104129	9.97
4796:E6	109444	3.84
4796:F6	113090	76.47
4796:G6	138429	12.02
4796:H6	142982	-6.91
4796:A7	149765	6.39
4796:B7	167410	-4.35
4796:C7	168597	27.37
4796:D7	170984	16.11
4796:E7	173904	21.74

4796:F7	174176	97.44
4796:G7	196524	15.60
4796:H7	256927	12.53
4796:A8	265473	7.93
4796:B8	267700	9.46
4796:C8	267712	34.53
4796:D8	273829	22.25
4796:E8	292663	16.62
4796:F8	293015	12.02
4796:G8	329696	15.60
4796:H8	330515	9.46
4796:A9	331757	12.53
4796:B9	345081	8.95
4796:C9	349644	9.97
4796:D9	351306	15.09
4796:E9	374898	63.68
4796:F9	376248	5.37
4796:G9	600300	43.73
4796:H9	603578	95.40
4796:A10	618261	3.32
4796:B10	620280	9.97
4796:C10	623746	50.90
4796:D10	624158	90.28
4796:E10	624947	60.10
4796:F10	626120	11.51
4796:G10	627666	28.90
4796:H10	634503	93.35
4796:A11	634926	36.57
4796:B11	634928	13.55
4796:C11	635312	7.42
4796:D11	635366	6.91
4796:E11	635436	11.00
4796:F11	635824	15.60
4796:G11	635833	11.00
4796:H11	637916	-4.35
4797:A2	639828	11.53
4797:B2	640974	-2.01
4797:C2	641228	36.59
4797:D2	641253	38.10
4797:E2	641607	2.51
4797:F2	642048	-2.01
4797:G2	643148	10.03
4797:H2	643599	-7.52
4797:A3	648419	3.06
4797:B3	650792	-7.52
4797:C3	657298	-0.50
4797:D3	657446	3.61
4797:E3	657598	5.61

4797:F3	657603	13.53
4797:G3	658293	6.27
4797:H3	658494	11.53
4797:A4	659174	3.03
4797:B4	662825	7.02
4797:C4	664181	5.01
4797:D4	668260	31.08
4797:E4	670224	9.02
4797:F4	670225	3.01
4797:G4	670226	-6.02
4797:H4	670229	1.03
4797:A5	673622	3.53
4797:B5	689228	2.51
4797:C5	689857	37.59
4797:D5	693172	80.20
4797:E5	697443	53.13
4797:F5	698031	2.58
4797:G5	7521	-5.51
4797:H5	7522	-7.52
4797:A6	14975	-11.03
4797:B6	18298	40.60
4797:C6	32992	3.51
4797:D6	34391	30.58
4797:E6	36354	5.01
4797:F6	45383	4.54
4797:G6	56817	3.61
4797:H6	79688	20.55
4797:A7	93419	9.05
4797:B7	96932	76.19
4797:C7	102815	-10.53
4797:D7	126728	12.03
4797:E7	129414	-6.02
4797:F7	132791	7.02
4797:G7	133071	2.01
4797:H7	139105	-6.52
4797:A8	672425	-5.01
4797:B8	337766	2.56
4797:C8	647418	14.54
4797:D8	164909	49.62
4797:E8	637993	2.03
4797:F8	534	98.75
4797:G8	4644	67.67
4797:H8	15623	-8.02
4797:A9	56737	3.01
4797:B9	106997	-1.50
4797:C9	183359	2.51
4797:D9	211489	46.12
4797:E9	605583	7.54

4797:F9	622586	26.57
4797:G9	622589	6.02
4797:H9	629738	51.13
4797:A10	638634	35.09
4797:B10	682769	51.13
4797:C10	10010	36.59
4797:D10	139490	4.51
4797:E10	48151	7.02
4797:F10	84074	-1.00
4797:G10	90829	-5.01
4797:H10	138925	-2.51
4797:A11	624206	33.58
4797:B11	118030	44.61
4797:C11	14229	7.57
4797:D11	90487	55.14
4797:E11	282880	-10.03
4797:F11	311153	31.58
4797:G11	643351	54.14
4797:H11	154890	58.15
4798:A2	164914	-6.17
4798:B2	165563	-10.00
4798:C2	167780	-0.64
4798:D2	169676	-7.45
4798:E2	172924	-8.30
4798:F2	173046	-5.74
4798:G2	174121	-10.43
4798:H2	177365	5.11
4798:A3	180973	9.79
4798:B3	184398	6.38
4798:C3	184403	7.02
4798:D3	240419	7.23
4798:E3	243928	-4.57
4798:F3	245432	-5.74
4798:G3	255109	-10.85
4798:H3	258812	-2.91
4798:A4	268251	-1.98
4798:B4	269148	-3.85
4798:C4	269754	-4.04
4798:D4	285223	-9.15
4798:E4	288010	-5.11
4798:F4	290205	23.62
4798:G4	305222	15.53
4798:H4	324368	-1.91
4798:A5	328166	-1.49
4798:B5	330500	6.17
4798:C5	330516	0.64
4798:D5	330753	-6.17
4798:E5	345647	-7.87

4798:F5	354844	-1.06
4798:G5	363744	0.21
4798:H5	363998	-6.17
4798:A6	603624	-5.11
4798:B6	617570	-5.74
4798:C6	623093	-1.28
4798:D6	623095	-3.53
4798:E6	635544	-3.40
4798:F6	638646	-2.57
4798:G6	640584	9.15
4798:H6	640637	3.19
4798:A7	640638	5.32
4798:B7	640985	2.77
4798:C7	641233	-2.13
4798:D7	642033	-5.32
4798:E7	644794	-4.47
4798:F7	653000	-1.13
4798:G7	654259	-2.17
4798:H7	657457	1.28
4798:A8	657722	-7.45
4798:B8	657799	4.47
4798:C8	658139	1.06
4798:D8	658350	6.17
4798:E8	659999	9.36
4798:F8	670140	6.17
4798:G8	671136	9.79
4798:H8	671394	7.45
4798:A9	679524	10.64
4798:B9	679527	6.81
4798:C9	681744	7.02
4798:D9	686349	18.94
4798:E9	687330	-10.64
4798:F9	690634	-1.06
4798:G9	699479	-3.40
4798:H9	703550	-2.13
4798:A10	4320	-1.91
4798:B10	5159	-2.98
4798:C10	7525	-6.81
4798:D10	7530	-4.89
4798:E10	19990	10.85
4798:F10	30916	24.47
4798:G10	46061	-9.36
4798:H10	65104	-8.30
4798:A11	68075	-5.11
4798:B11	70845	-2.77
4798:C11	70929	7.87
4798:D11	73495	-2.19
4798:E11	76455	-2.19

4798:F11	93135	-6.60
4798:G11	103248	-1.26
4798:H11	109350	-3.85
4799:A2	116693	2.10
4799:B2	136044	-7.13
4799:C2	145366	-9.22
4799:D2	153858	-6.77
4799:E2	156215	-7.97
4799:F2	169774	-1.26
4799:G2	208734	-6.29
4799:H2	218439	-1.29
4799:A3	243023	-1.32
4799:B3	248436	4.19
4799:C3	265450	-5.16
4799:D3	268242	0.42
4799:E3	274893	4.19
4799:F3	281613	7.13
4799:G3	304421	2.94
4799:H3	328426	0.84
4799:A4	332598	-0.84
4799:B4	337851	13.00
4799:C4	353527	11.74
4799:D4	359463	-1.74
4799:E4	600681	0.84
4799:F4	607316	-2.10
4799:G4	619907	49.06
4799:H4	620261	-1.68
4799:A5	624953	-5.09
4799:B5	625331	8.39
4799:C5	625873	-7.55
4799:D5	640335	40.67
4799:E5	645567	3.77
4799:F5	653558	4.61
4799:G5	669356	0.84
4799:H5	676963	20.96
4799:A6	677392	-3.06
4799:B6	688363	3.77
4799:C6	7532	3.77
4799:D6	24559	-2.22
4799:E6	34931	-1.38
4799:F6	49842	3.77
4799:G6	58514	-5.58
4799:H6	67574	-0.42
4799:A7	76027	64.15
4799:B7	90636	2.10
4799:C7	107412	1.26
4799:D7	115493	1.68
4799:E7	226080	-2.10

4799:F7	253995	5.03
4799:G7	269146	-6.00
4799:H7	306864	-2.94
4799:A8	325319	2.10
4799:B8	363182	5.03
4799:C8	526417	-5.45
4799:D8	603169	2.94
4799:E8	638352	-1.09
4799:F8	640342	-7.44
4799:G8	643834	4.19
4799:H8	697468	-1.74
4799:A9	3053	-2.48
4799:B9	18268	-9.15
4799:C9	361813	5.45
4799:D9	641250	9.22
4799:E9	71948	-5.15
4799:F9	683792	2.94
4799:G9	675593	18.87
4799:H9	181486	2.94
4799:A10	192965	3.35
4799:B10	219734	-1.09
4799:C10	700582	-2.94
4799:D10	52141	-3.77
4799:E10	166454	1.26
4799:F10	97338	-10.06
4799:G10	260610	-6.29
4799:H10	267229	-7.13
4799:A11	276299	-6.71
4799:B11	641240	13.00
4799:C11	641245	-2.10
4799:D11	642040	1.68
4799:E11	239375	13.00
4799:F11	292567	1.68
4799:G11	125066	-3.77
4799:H11	265211	-9.64
4800:A2	125176	-5.36
4800:B2	285116	-2.56
4800:C2	333856	-1.17
4800:D2	622116	5.83
4800:E2	622124	90.21
4800:F2	39863	53.38
4800:G2	636126	2.10
4800:H2	49451	-0.70
4800:A3	645617	-5.36
4800:B3	655255	3.50
4800:C3	65423	6.76
4800:D3	72961	4.90
4800:E3	102811	15.62

4800:F3	165897	5.83
4800:G3	305819	1.17
4800:H3	615593	55.71
